# Fosmetpantotenate (RE-024), a phosphopantothenate replacement therapy for pantothenate kinase-associated neurodegeneration: Mechanism of action and efficacy in nonclinical models

**DOI:** 10.1371/journal.pone.0192028

**Published:** 2018-03-09

**Authors:** Daniel Elbaum, Maria G. Beconi, Edith Monteagudo, Annalise Di Marco, Maria S. Quinton, Kathryn A. Lyons, Andrew Vaino, Steven Harper

**Affiliations:** 1 Research and Development, Retrophin Inc., Cambridge, Massachusetts, United States of America; 2 Preclinical Research, IRBM Science Park SpA, Pomezia, Rome, Italy; 3 *In-vitro* Pharmacology, IRBM Science Park SpA, Pomezia, Rome, Italy; 4 Independent consultant, Holland, New York, United States of America; 5 Medicinal Chemistry, IRBM Science Park SpA, Pomezia, Rome, Italy; Hungarian Academy of Sciences, HUNGARY

## Abstract

In cells, phosphorylation of pantothenic acid to generate phosphopantothenic acid by the pantothenate kinase enzymes is the first step in coenzyme A synthesis. Pantothenate kinase 2, the isoform localized in neuronal cell mitochondria, is dysfunctional in patients with pantothenate kinase-associated neurodegeneration. Fosmetpantotenate is a phosphopantothenic acid prodrug in clinical development for treatment of pantothenate kinase-associated neurodegeneration, which aims to replenish phosphopantothenic acid in patients. Fosmetpantotenate restored coenzyme A in short-hairpin RNA pantothenate kinase 2 gene-silenced neuroblastoma cells and was permeable in a blood-brain barrier model. The rate of fosmetpantotenate metabolism in blood is species-dependent. Following up to 700 mg/kg orally, blood exposure to fosmetpantotenate was negligible in rat and mouse, but measurable in monkey. Consistent with the difference in whole blood half-life, fosmetpantotenate dosed orally was found in the brains of the monkey (striatal dialysate) but was absent in mice. Following administration of isotopically labeled-fosmetpantotenate to mice, ~40% of liver coenzyme A (after 500 mg/kg orally) and ~50% of brain coenzyme A (after 125 μg intrastriatally) originated from isotopically labeled-fosmetpantotenate. Additionally, 10-day dosing of isotopically labeled-fosmetpantotenate, 12.5 μg, intracerebroventricularly in mice led to ~30% of brain coenzyme A containing the stable isotopic labels. This work supports the hypothesis that fosmetpantotenate acts to replace reduced phosphopantothenic acid in pantothenate kinase 2-deficient tissues.

## Introduction

Pantothenate kinase-associated neurodegeneration (PKAN) is a rare autosomal recessive neurodegenerative disease [1–3] caused by a mutation in the pantothenate kinase-2 (*PANK2*) gene on chromosome 20p13. More than 100 mutations have been published [4], including missense, nonsense, splice-site, and frameshift mutations [5]. PKAN manifests as a range of signs of parkinsonism, dystonia, and other systemic features. The clinical presentation, age of onset, and rate of progression are highly variable, even among siblings and case clusters with identical mutations [5–9], leading to a poor understanding of the genotype/phenotype relationship [10].

In PKAN, the mutation in the *PANK2* gene, encoding the mitochondrial form of pantothenate kinase (PanK), causes a reduction in the activity of the PanK2 enzyme, which converts pantothenate (PA, vitamin B5) to phosphopantothenate (PPA), in the biosynthetic pathway to coenzyme A (CoA) [11]. It has been postulated that the defective PanK2 enzyme, localized primarily in mitochondria [1], has decreased ability to phosphorylate pantothenate, leading to decreased concentrations of CoA in vulnerable tissues, including brain. CoA and its acetylated metabolite, acetyl-CoA, are key factors in several cellular processes, including energy metabolism, autophagy, and mitosis, acting as a central metabolic intermediate and as signal transducers in anabolic and catabolic reactions [12].

**Fig 1:**
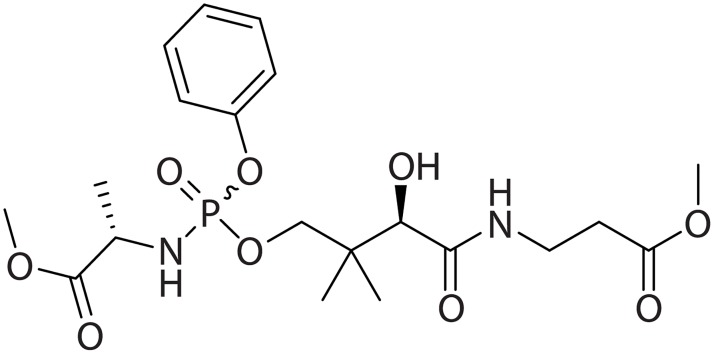
Structure of fosmetpantotenate.

Currently, there is no disease-modifying or specific therapy for patients with PKAN. Fosmetpantotenate (RE-024, Fig 1) is under development as a PPA replacement therapy to treat patients with PKAN. The U.S. Food and Drug Administration (FDA) granted fosmetpantotenate both orphan drug status and fast track designation, and a pivotal clinical trial has started (NCT03041116, https://www.clinicaltrials.gov/ct2/show/NCT03041116?term=03041116&rank=1) [13, 14].

**Fig 2:**
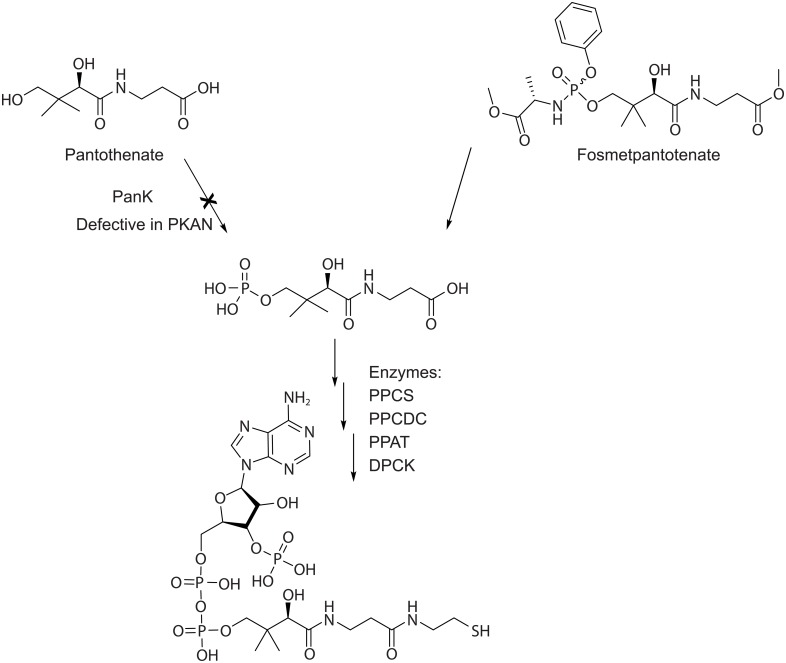
Mechanism of action postulated for fosmetpantotenate. DPCK: dephospho-CoA kinase; PPAT: 4’-phosphopantetheine adenylyltransferase; PPCDC: (R)-4’-phospho-N-pantothenoylcysteine decarboxylase; PPCS: 4’-phosphopantothenoylcysteine synthetase.

It has been reported that 4'-phosphopantothenic acid is not permeable to cell membranes [2] due to its anionic character, consistent with the observation that systemic administration of PPA does not restore CoA levels in cellular and mouse models of PKAN [15]. As such, fosmetpantotenate is a 4'-phosphopantothenic acid (ie, PPA) precursor designed to deliver PPA to cells, replenishing the product of dysfunctional PanK (Fig 2). The use of prodrug strategies in the field of phosphate drugs has been explored previously [16]. By masking the charge of the phosphate dianion, the membrane permeability of fosmetpantotenate is increased as compared to PPA, and once in cells, it can be metabolized to PPA, becoming a substrate for downstream CoA-generating enzymes in the same manner as endogenously produced PPA.

Fosmetpantotenate masks PPA with a methyl ester at the PA carboxylate, a phenol phosphoester, and an alanine methyl ester phosphoramidate. Fosmetpantotenate is a colorless oil containing two chiral carbon atoms with known absolute stereochemistry. The amino acid portion of the molecule is prepared from naturally occurring L-alanine. The secondary alcohol functionality is part of naturally occurring vitamin B5 and has R-stereochemistry. The phosphorus atom is also a chiral center, making fosmetpantotenate a mixture of two diastereomers.

This report describes the studies we conducted to explore the mechanism of action of fosmetpantotenate. Initially, we evaluated its efficacy in vitro using the human neuronal cell line IMR32, in which we specifically downregulated the expression of PanK2 using a short-hairpin RNA (shRNA) directed at the *PANK2* gene product (Supplementary Information S1 Fig). Findings in this cell model demonstrated that fosmetpantotenate is effective in restoring CoA levels and in rescuing defects in tubulin acetylation, a CoA-dependent process. Furthermore, in vivo studies show that once fosmetpantotenate reaches a given tissue, PPA derived specifically from the compound can be incorporated into CoA.

Finally, cross-species findings suggest that, due to large differences in systemic rates of metabolism, mice and rats are not expedient animal models to evaluate brain penetration of fosmetpantotenate.

## Results

### In vitro effects of fosmetpantotenate following incubations in shRNA PanK2 knocked down human neuroblastoma cells

**Fig 3:**
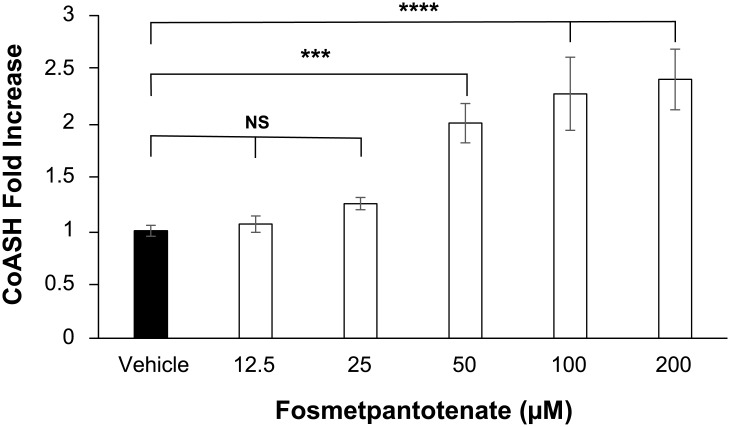
Intracellular CoA concentrations in shRNA PanK2 knockdown human neuroblastoma cells following incubations with fosmetpantotenate. Experiment in triplicate. One-way ANOVA with Dunnett’s post-hoc analysis; *** p ≤0.001 **** p≤0.0001. NS: not significant.

**Fig 4:**
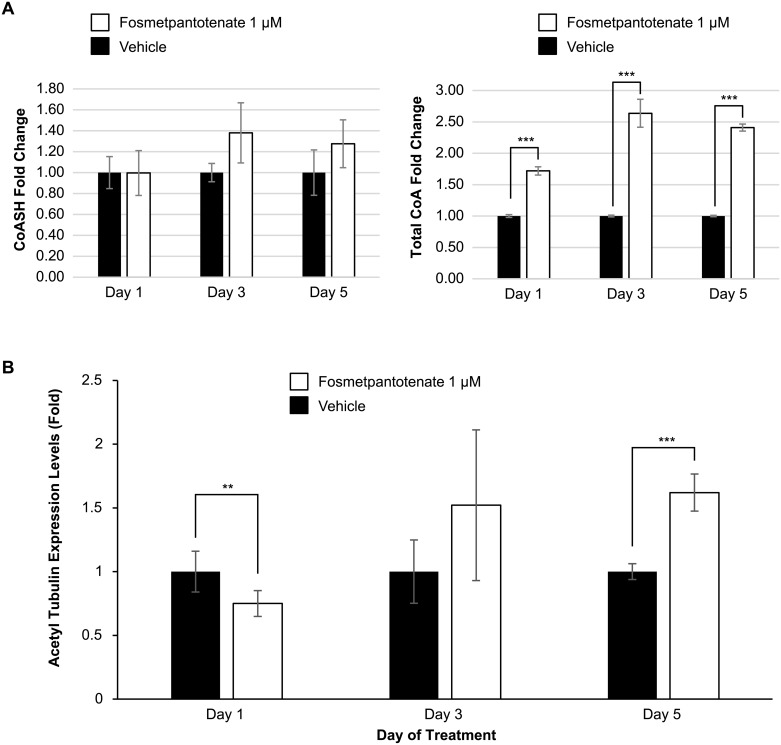
Effects of 1 μM fosmetpantotenate TID for 5 consecutive days in shRNA PanK2 knockdown human neuroblastoma cells. (A) Intracellular CoA concentrations, n = 3. (B) Western blot densitometry values. β-actin was used for normalization. Two experiments in duplicate. Two sided t-test; *p ≤0.05, **p ≤0.01, ***p ≤0.001. Gel images can be found in S2 Fig.

Our initial experiments were designed to characterize the effects of fosmetpantotenate on shRNA PanK2 knocked down cells. To this end we treated cells with varying concentrations of fosmetpantotenate (12.5, 25, 50, 100, and 200 μM) for 2 days (to maintain consistency with early assays in this program, the medium with fresh compound was changed at 24 h). These conditions resulted in a 2- to 4-fold increase in free CoA levels (Fig 3). While this finding was encouraging, the compound concentrations were higher than can be expected in a clinical setting. Thus, we also treated these cells with 1 μM fosmetpantotenate in a three-times-daily (TID) regime for 5 days, to mimic the anticipated clinical dosing schedule. In this case, we observed little change in the level of free CoA but did observe a 1.6- to 2.6-fold increase in total CoA during this experiment (Fig 4A). Further analysis showed that the ratio of total CoA to free CoA was ~6- to 7-fold. Over the course of our PKAN program, we have found this ratio to be approximately 4-fold (data not shown).

In a separate experiment, Pank2 knockdown cells were treated as above, and the level of acetyl tubulin was measured on days 1, 3, and 5 (Fig 4B). There was a clear trend toward increasing acetyl tubulin over the course of the experiment, with a statistically significant ~1.6-fold increase observed on day 5. Unexpectedly, there was a statistically significant decrease on day 1. We can only speculate as to why this occurred, as the effects of restoring CoA after deprivation are just beginning to be explored.

When treated acutely with 25, 50, or 200 μM fosmetpantotenate once daily (QD) for 2 days, a 2- to 5-fold increase in tubulin acetylation levels was observed in these cells (Table 1). The similarity of the tubulin acetylation levels observed with 25 to 200 μM fosmetpantotenate incubations compared to those achieved after 5 days of dosing the cells with fosmetpantotenate at 1 μM TID indicates that low-dose treatment over an extended time period recapitulates the effects seen upon acute treatment with a high dose.

**Table 1:** Tubulin acetylation levels (fold of PanK2 control knockdown) 24 h following incubations of fosmetpantotenate at 25, 50, or 200 μM concentrations.

Treatment	Tubulin Acetylation (fold of control)^#^
PanK2 KD vehicle	1
Fosmetpantotenate 25μM	2.32 ± 0.64
Fosmetpantotenate 50 μM	3.68 ± 1.13
Fosmetpantotenate 200 μM	5.12 ± 2.57

^#^ Means ± standard deviation. N = 3 independent experiments with the following replicates: experiment 1 n = 1, experiment 2 n = 3 at 50 and 200 μM, experiment 3 n = 3 at all dose levels. β-actin was used for normalization.

#### In vitro metabolism

Fosmetpantotenate and its individual diastereomers (D1 and D2) were stable (half-life [t½] >10 h) when incubated in simulated gastric fluid (pH 1.2; 3.2 mg/mL pepsin) at 10 μM. These data indicate that fosmetpantotenate would undergo negligible degradation in the stomach and that a large fraction of the dose would be available for absorption after oral (PO) administration.

Fosmetpantotenate diastereomers D1 and D2 have poor stability in mouse and rat blood (5 μM, 37°C) (Table 2), with t½ <5 min for the mixture or individual diastereomers. Fosmetpantotenate was somewhat more stable in incubations in monkey and human blood (Table 2). These in vitro data indicate that fosmetpantotenate is rapidly metabolized by blood enzymes and that the rate of metabolism is species-dependent. The <5 min t½ predicts that fosmetpantotenate will have a very short t½ in mouse and rat circulation. In the monkey and human, the in vitro data predict an in vivo t½ of 10 to 45 min.

**Table 2:** Mean half—Life of fosmetpantotenate and diastereomers after incubation with blood from various species at 37°C for 60 min^a^.

Mean half-life (min)			
Species	RE-024	D1	D2
Mouse	<5	<5	<5
Rat	<5	<5	<5
Monkey	41	35	17
Human	67	44	>95

^a^ Experiment run in duplicate

Fosmetpantotenate was unstable when incubated in liver microsomes, with half-lives of 11, 8, 11, and 21 min, respectively, in mouse, rat, monkey, and human liver microsomes. The corresponding intrinsic clearances of 247, 335, 263, and 134 μL/min/mg microsomal protein, respectively, predict very high metabolic hepatic extraction.

#### Blood-brain barrier permeability

The blood-brain barrier permeabilities of fosmetpantotenate, its isolated diastereomers D1 and D2, PA, and PPA were assessed in vitro in a two-dimensional non-contact porcine brain endothelial cell (PBEC) co-culture model adapted from Patabendige and co-workers [17, 18]. This model retains the inductive influence from astrocytes of endothelial cell barrier function and expresses key proteins associated with intercellular tight junctions and major blood-brain barrier drug transporters. In addition, this model has been validated by measuring the apparent in vitro permeability of more than 20 structurally diverse compounds selected based on the availability of in situ brain perfusion data from rat studies [19–21]. The rank order of the permeability of these compounds was comparable with that of the in situ brain penetration measured in rats (data not shown).

The apparent in vitro permeability values measured in this model for fosmetpantotenate, D1, D2, PA, and PPA are reported in Table 3. The permeability of reference compounds with known mechanisms of brain penetration was also determined and reported for comparison. The permeability of diastereomers of fosmetpantotenate was about 2- to 3-fold higher than compounds used as markers for paracellular permeability/passive diffusion (sucrose and mannitol), while PPA was relatively impermeable. Permeability values of D1 and D2 indicate that they can be classified as moderately penetrant in the blood-brain barrier model, with mannitol and sucrose at the low end of permeability range, S-3,4-dihydroxyphenylalanine (L-DOPA) in the moderate permeability range, and naloxone at the high permeability range.

**Table 3:** Apparent in vitro permeability of diastereomers of fosmetpantotenate, PA, and PPA in a blood—Brain barrier permeability model using co-cultured porcine brain endothelial cells and rat astrocytes^a^.

Compound	In Vitro Measured P_app_ (10^−6^ cm/s)	TEER (Ω × cm^2^)
Sucrose	1.6 ± 0.5	1032 ± 52
Mannitol	2.2 ± 0.7	779 ± 146
Naloxone	42.3 ± 11.0	1303 ± 14
Caffeine	34.3 ± 3.1	789 ± 173
L-DOPA	5.8 ± 1.9	715 ± 188
PA	4.2 ± 1.3	746 ± 80
D1	4.8 ± 1.8	553 ± 40
D2	4.0 ± 1.3	553 ± 40
PPA	1.0 ± 0.3	939 ± 46

^a^ Experiments run in triplicate

Values represent the mean ± SD (n = 3). Incubations were considered acceptable when the apparent permeability (P_app_) of the paracellular marker FITC-40 kDa was <1×10^−6^ cm/s and recovery of tested compound ≥80%. The P_app_ of the paracellular marker sucrose was 3.61 ± 1.74. Sucrose is a known blood-brain barrier permeability marker [22]. P_app_: apparent permeability; SD: standard deviation; TEER: transepithelial electrical resistance

#### Pharmacokinetics in whole blood

Consistent with the rapid in vitro metabolism, fosmetpantotenate was not detected in significant amounts in blood from mice or rats at PO doses up to 700 mg/kg (Table 4).

**Table 4:** Blood pharmacokinetics of fosmetpantotenate, PPA, and PA in male mice, rats, and monkeys following a single PO dose of fosmetpantotenate^a^.

Analyte	Fosmetpantotenate		PPA		PA	
PO Dose (mg/kg)	C_max_ (nM)	AUC (nM*h)	C_max_ (nM)	AUC (nM*h)	C_max_ (nM)	AUC (nM*h)
Mouse (CD-1)						
control	<LLOQ	NC	<LLOQ	NC	382^b^	2,260^b^
100	7.8	4.06	521	260	683^c^	3,400^c^
300	30.2	6.70	2,590	1,330	771^c^	5,760^c^
700	307	123	7,590	3,250	1,610^c^	10,500^c^
Rat (Sprague-Dawley)						
control	<LLQO	NC	<LLOQ	NC	2280^b^	14300^b^
100	NC	NC	4,130	3,870	1,340^c^	8,680^c^
300	290	120	23,900	21,600	4,750^c^	32,500^c^
700	1,290	1,920	20,200	19,800	7,900^c^	40,100^c^
Monkey (Cynomolgus)						
300	18,700 (9950, 27400)	34,400 (13000, 55800)	323 (275, 371)	1550 (1140, 1960)	589^d^ (483, 695)	3350^d^ (2930, 3760)

^a^ PK parameters were calculated using the average concentration per time point for mouse (N = 4) and rat (N = 3) and the average of individual PK parameters (N = 2, individual values in parentheses) for monkeys.

^b^ PA PK parameters from control (vehicle) dosed mouse and rat cohort are based on total concentration in blood and represent basal levels (N = 2 per time point).

^c^ Reported PA PK parameters for mouse and rat groups dosed with fosmetpantotenate were adjusted for pre-dose PA.

^d^ Reported PA PK parameters were adjusted for pre-dose PA concentrations 26 and 49 nM.

LLOQ for fosmetpantotenate: mouse and rat = 1.02 nM. LLOQ for PPA: mouse and monkey = 20.0 nM, rat: = 10 nM. NC: not determined due to insufficient values >LLOQ; PO: oral

**Fig 5:**
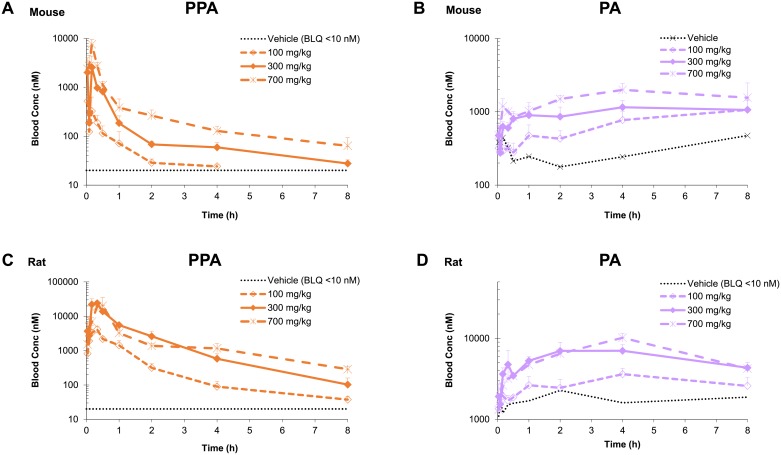
PPA and total PA in mouse and rat blood. Concentration-versus-time plots of PPA and PA after a single oral administration of fosmetpantotenate at 100, 300, or 700 mg/kg in CD1 mice (N = 4 per time point) or Sprague Dawley rats (N = 3 per time point).

For both mice and rats, several metabolites of fosmetpantotenate containing the PPA moiety were present in circulation (Table 5). The concentrations of the end products of fosmetpantotenate metabolism, PPA and PA, increased in a dose-related manner following fosmetpantotenate administration (Table 4, Fig 5). For PPA, as dose increased from 100 to 700 mg/kg, mouse maximum concentration (C_max_) and area under the concentration-versus-time curve (AUC) increased 15- and 12-fold, respectively, and rat C_max_ and AUC increased 4.9- and 5.1-fold, respectively. For PA, basal pharmacokinetic (PK) parameters were estimated based on concentrations from a cohort dosed orally with vehicle; C_max_ and AUC are reported in Table 4. Following dosing at 100 mg/kg of fosmetpantotenate, mouse basal corrected PA C_max_ was 683 nM and AUC was 3400 nM*h. As dose increased to 700 mg/kg, mouse basal adjusted C_max_ and AUC increased 2.4- and 3.1-fold, respectively, while in rats dosed at 100 mg/kg, basal adjusted C_max_ was 1340 nM and AUC was 8680 nM*h. As dose increased to 700 mg/kg, rat basal adjusted C_max_ and AUC increased 5.9- and 4.6-fold, respectively.

**Table 5:** Area under the curve for circulating metabolites as a percentage of fosmetpantotenate (parent) after a single oral administration of 100, 300, or 700 mg/kg to mice, rats, and monkeys.

Metabolite	Dose (PO mg/kg)	% Parent AUC_All_^a^		
		CD1 Mouse	Sprague-Dawley Rat	Cynomolgus Monkey
Compound 1	100	129%	ND^b^	NT^c^
	300	307%	39%	19%
	700	257%	22%	NT
Compound 2	100	8,550%	ND	NT
	300	29,800%	1,250%	132%
	700	7,220%	220%	NT
Compound 3	100	8,900%	ND	NT
	300	18,900%	4,130%	9%
	700	1,990%	209%	NT
Compound 4	100	3,180%	ND	NT
	300	12,600%	2,820%	301%
	700	10,500%	849%	NT

^a^ % fosmetpantotenate (Parent) AUC = metabolite AUC_all_/Parent AUC_all_ x 100

^b^ ND, not determined. AUC of parent was not determined at the 100 mg/kg PO dose in rats due to insufficient fosmetpantonate values >LLOQ.

^c^ NT, not tested.

These data further support the hypothesis that fosmetpantotenate is well absorbed but rapidly converted to PPA and PA, consistent with the in vitro data (Table 2).

**Fig 6:**
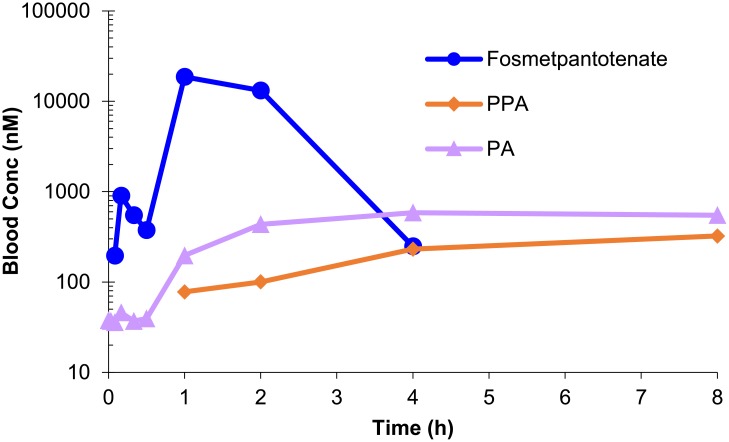
Fosmetpantotenate, PPA, and total PA in monkey blood. Concentrations of fosmetpantotenate, PPA, and PA after a single oral administration of fosmetpantotenate in cynomolgus monkeys at 300 mg/kg (N = 2).

**Fig 7:**
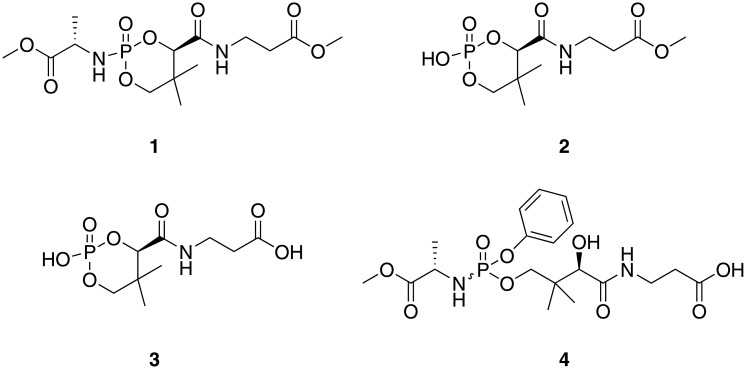
Four PPA-containing metabolites of fosmetpantotenate detected in monkey blood after a single oral administration of 300 mg/kg.

In monkeys, fosmetpantotenate was quantifiable in blood. After an oral dose of 300 mg/kg (N = 2), absorption was rapid (time to maximum concentration [T_max_] = 1 h), reaching mean C_max_ of 18,700 nM and AUC of 34,000 nM*h. Individual exposure varied by 3- to 4-fold (see Table 4 for individual values). However, that extent of variation would not be unusual for low to moderately bioavailable compounds where bioavailability for the two aforementioned subjects was determined in a crossover study as 11% and 37% (i.v. data not reported). The concentrations of PPA and PA increased with fosmetpantotenate administration (Table 4, Fig 6). In the case of PPA, concentrations increased from pre-dose levels of below lower limit of quantitation (LLOQ <20 nM) to an observed C_max_ of 323 nM at 8 h. Average basal pre-dose levels of PA were 37 nM and increased to a C_max_ of 589 nM at 8 h. Additional fosmetpantotenate metabolites containing the PPA moiety were present in circulation and represented a significant percentage of the fosmetpantotenate AUC (Table 5, Fig 7).

#### Pharmacokinetics in brain dialysate

**Fig 8:**
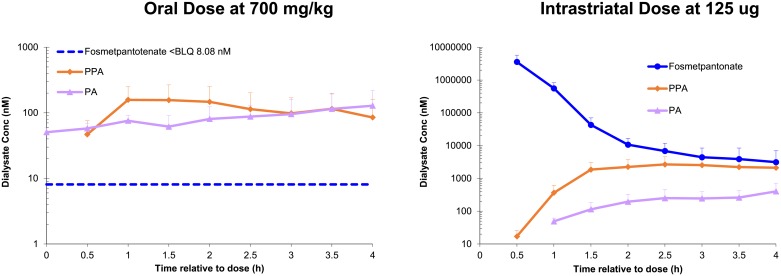
Fosmetpantotenate, PPA, and total PA in mouse brain striatal dialysate. Single administration of fosmetpantotenate in C57Bl6 mice (700 mg/kg orally or 125 μg intrastriatally).

In mice following a 700 mg/kg oral dose, fosmetpantotenate and most PPA-containing metabolites were not detected in dialysate (data not shown). Concentrations of PA and PPA were measurable: PPA reached a C_max_ of 177 nM at 1.4 h. PA exhibited an average baseline (pre-dose) concentration of 51 nM and reached a basal adjusted C_max_ of 88 nM (total PA C_max_ of 130 nM) at 3.8 h (Table 6, Fig 8). In contrast to the oral dose, when fosmetpantotenate was administered intrastriatally, fosmetpantotenate was detected at high concentrations in the dialysate, with a C_max_ of 3.6 x 10^6 nM at 0.5 h. PPA reached a C_max_ of 2980 nM at 3.0 h, and PA C_max_ was 405 nM at 3.9 h. Although both PA and PPA were detected in pre-dose dialysate in the intrastriatal dosed cohort, concentrations were either low or <LLOQ in most samples; hence, no adjustment was made for baseline contribution to PPA or PA PK parameters (Table 6, Fig 8). In contrast, isotopically labeled CoA was detected in brain tissue when isotopically labeled fosmetpantotenate was used (see below in Mechanism of Action section).

**Table 6:** Estimated blood and dialysate pharmacokinetics of fosmetpantotenate, PPA, and PA in male mice after a single dose of fosmetpantotenate at 700 mg/kg PO or 125 μg intrastriatally and in monkeys following a single PO dose of fosmetpantotenate at 100 or 300 mg/kg^a^.

Analyte		Fosmetpantotenate		PPA^b^			PA^c^		
Fosmetpantotenate Dose	Matrix	C_max_ (nM)	AUC (nM*h)	Baseline conc (nM)^d^	C_max_ (nM)^b^	AUC (nM*h)^b^	Baseline conc (nM)^d^	C_max_ (nM)	AUC (nM*h)
Mouse (C57Bl6)									
700 mg/kg PO	Dialysate	<LLOQ	NC	<LLOQ	177 ± 97	426 ± 302	50.7 ± 8.2	88.0 ± 74.3	154 ± 105
125 μg IS		3.57 ± 2.19 x10^^6^	2.10 ± 1.24 x10^^6^	13 + 6^e^	2980 ± 1920^e^	6490 ± 4150^e^	23^f^	405 ± 306^f^	648 ± 440^f^
Monkey (Cynomolgus)									
100 mg/kg PO	Blood	1650 ± 1930	1870 ± 1960	<LLOQ	151 ± 68	216 ± 50	56 ± 26	255 ± 70	467 ± 152
300 mg/kg PO		6040 (7390, 4690)	6810 (8530, 5090)	<LLOQ	2900 (2930, 2880)	4720 (3500, 5950)	184 (123, 245)	4270 (4210, 4340)	9340 (9920, 8760)
100 mg/kg PO	Dialysate	221±253	209 ± 234	32 ±14	593 ± 343^g^	1240 ± 726^g^	276 ± 276	432 ± 221	816 ± 374
300 mg/kg PO		1560 (2060, 1060)	1030 (1270, 790)	ND	ND	ND	944 (766, 1120)	1300 (1440, 1160)	2170 (1970, 2380)

^a^ Due to the short duration of the study, PK parameters are considered estimates. AUC values represent mean ± standard deviation for mice (700 mg/kg PO n = 8, 125 μg IS n = 7) and 100 mg/kg dosed monkeys (n = 4), and mean (individual values) for 300 mg/kg dosed monkeys.

^b^ PPA parameters were calculated using total PPA concentrations and were adjusted for pre-dose levels only for monkeys at 100 mg/kg. For all other dose/species combinations, PPA concentrations were not adjusted for baseline concentrations since PPA pre-dose levels were <LLOQ.

^c^ With the exception of the mouse dialysate following intrastriatal administration where PA concentrations were <LLOQ, PA PK parameters were calculated using concentrations adjusted for pre-dose levels.

^d^ Baseline concentration was the mean concentration in pre-dose samples.

^e^ PPA and PA concentrations were not adjusted for pre-dose concentrations, since the majority of the pre-dose levels were <LLOQ.

^f^ PPA and PA concentrations were not adjusted for pre-dose concentrations, since the majority of the pre-dose levels were <LLOQ.

^g^ PPA PK parameters were adjusted for pre-dose concentrations.

Fosmetpantotenate LLOQ: mouse and rat dialysate = 8.10 nM, monkey blood = 1.50 nM, monkey dialysate = 0.192 nM (100 mg/kg study) and 0.550 nM (300 mg/kg study). PPA LLOQ: mouse and rat dialysate = 2.70 nM, monkey blood = 15.0 nM (100 mg/kg study) and 12.0 nM (300 mg/kg study), monkey dialysate = 3.06 nM (100 mg/kg study). PA LLOQ: mouse and rat dialysate = 17.5 nM, monkey blood = 30.0 nM (100 m/kg study) and 72.0 nM (300 mg/kg study), monkey dialysate = 3.06 nM (100 mg/kg study) and 6.19 nM (300 mg/kg study). IS, intrastriatal; ND: Not determined due to insufficient sample volume for LC/MS analysis; PO, oral

**Fig 9:**
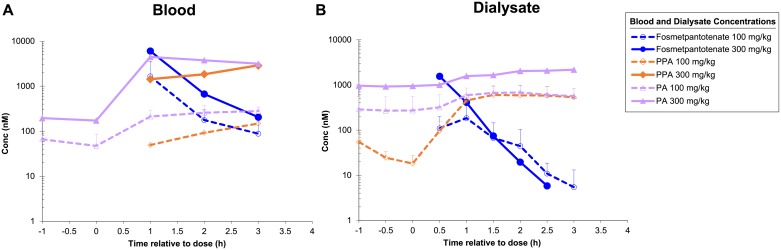
Fosmetpantotenate, PPA, and total PA in monkey blood and brain striatal dialysate. Single oral administration to cynomolgus monkeys (100 and 300 mg/kg).

The ability of fosmetpantotenate and metabolites to distribute to the brain was evaluated in vivo from striatal dialysate collections in monkey (Table 6, Fig 9 monkey). For these studies, animals were dosed orally with fosmetpantotenate, and blood and dialysate were collected from 1 h pre-dose to 3 h post fosmetpantotenate dose. C_max_ and AUC from time 0 to the last measurable time point were calculated to facilitate comparison of exposure. However, these values should be considered an estimate due to the short duration of the sample collection. Baseline levels of PA and PPA were estimated using averages from pre-dose concentrations. For those cases where pre-dose concentrations were >LLOQ, the corresponding reported C_max_ and AUC were adjusted for baseline contribution by subtracting the mean of pre-dose sample concentrations from post-dose concentrations per subject.

In contrast to the experiments in mice, when monkeys were dosed orally at either 100 or 300 mg/kg, fosmetpantotenate was detected in the brain dialysate. Both fosmetpantotenate dialysate C_max_ and AUC increased more than dose proportionally, with a 5.5-fold increase for C_max_ (221 nM at 0.75 h to 1560 nM at 0.4 h) and a 5.0-fold increase in AUC (209 nM*h to 1030 nM*h). In blood, fosmetpantotenate estimated exposure increased closer to dose proportional, ~3.6-fold. These data indicate that with increasing dose, brain exposure may increase in a greater than dose proportional manner (Table 6).

Baseline levels of PA were detected in all pre-dose blood and dialysate samples. While consistent within each dose group, there was approximately a 3.2-fold difference between the 100 mg/kg cohort and the 300 mg/kg cohort in basal PA levels in both blood (56 nM vs 184 nM) and dialysate (276 nM vs 944 nM) levels. Differences of this magnitude between animals are not uncommon, necessitating the adjustment for basal exposure to be done on an individual basis. As the dose increased from 100 to 300 mg/kg, there were dose proportional increases in PA dialysate basal adjusted C_max_ of 3.0-fold (432 nM vs 1300 nM) and AUC of 2.7-fold (816 nM*h vs 2170 nM*h). Dialysate PPA following the 100 mg/kg dose exhibited a C_max_ of 593 nM at 3 h, and the AUC was 1240 nM*h. PPA levels were not determined from the 300 mg/kg dose samples due to analytical issues. (Repetition of the experiment was precluded on ethical grounds.) Based on the fact that levels of both PA and PPA in blood increase in a dose proportional fashion, it would be expected that PPA levels in the brain dialysate would also increase.

Although blood levels of fosmetpantotenate peak quite early after an oral dose in both mice and monkeys, the levels of PA and PPA in both blood and brain dialysate continue to rise for several hours. This intriguing observation may be due to the presence of additional metabolites that contain the PPA moiety. The full metabolic pathway and PK of fosmetpantotenate will be published elsewhere.

### Mechanism of action

**Fig 10:**
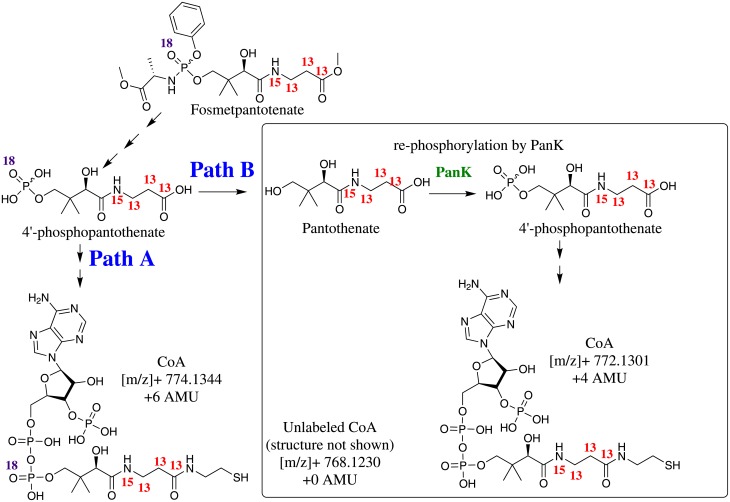
Scheme depicting the different metabolic paths to CoA formation from either pantothenic acid or fosmetpantotenate.

Successful incorporation of the PPA moiety contained within fosmetpantotenate was demonstrated following administration of isotopically-labeled fosmetpantotenate to wild type (WT) mice. Fosmetpantotenate was labeled with three ^13^C and one ^15^N in the PA moiety and with one ^18^O in the phosphate moiety, as indicated in Fig 10. The premise of this experiment is the following: if PPA incorporated into CoA originates from fosmetpantotenate metabolism, then all isotope labels (including the ^18^O) will be retained in the CoA molecule, resulting in an increase in molecular weight of 6 atomic mass units (AMU) with respect to unlabeled CoA ([M+H]^+^ = 774.3 vs 768.3, respectively; Fig 10, **Path A**). However, if fosmetpantotenate is hydrolyzed to PA, and PA is then re-phosphorylated by WT PanK to yield PPA, or if the ^18^O is exchanged with water, then the ^18^O would be lost in the process, resulting in a CoA molecule with a 4-AMU increase in molecular weight over unlabeled CoA ([M+H]^+^ = 772.3 vs 768.3, respectively, Fig 9, **Path B**). CoA derived from endogenous PA/PPA will have the unchanged molecular weight ([M+H]^+^ = 768.3).

Six hours after a 500 mg/kg single oral administration of isotopically labeled fosmetpantotenate to mice, 40% of the total CoA in liver samples contained PPA derived from the fosmetpantotenate molecule (+6 AMU), 1.4% contained fosmetpantotenate-PA that had been re-phosphorylated or where the ^18^O was lost by exchange (+4 AMU), and 58.6% of the CoA contained endogenous PPA. At 24 h post-dose, the percentage of CoA derived from fosmetpantotenate-PPA was slightly lower (34%), while the percentage of CoA derived from fosmetpantotenate-PA that had been re-phosphorylated by PanK (or oxygen lost by exchange) had increased slightly (8%). As expected from the rapid metabolism of fosmetpantotenate in mouse blood, incorporation of fosmetpantotenate-derived PPA in brain CoA was not observed (Table 7).

**Table 7:** Percentage of CoA derived from endogenous phosphopantothenic acid (unlabeled), fosmetpantotenate-derived pantothenic acid with rephosphorylation by WT PanK (+4 AMU), and fosmetpantotenate-derived phosphopantothenic acid (+6 AMU) following PO and intrastriatal administration of isotopically-labeled fosmetpantotenate to WT C57Bl6 mice^a^.

	500 mg/kg PO Liver CoA (%)			125 μg intrastriatally Brain CoA (%)		
Time (h)	Unlabeled	+4 AMU	+6 AMU	Unlabeled	+4 AMU	+6 AMU
**Pre-dose**	100	0	0	100	0	0
**6**	58.6	1.4	40	61	2.8	36.2
**12**	ND	ND	ND	50	3.6	46.4
**24**	58	8.4	34	45	4.3	50.7

^a^N = 3 mice per time point for PO experiment and n = 8 mice per time point for IS, pooled samples.

However, when fosmetpantotenate was dosed to mice intrastriatally (125 μg), 36.2% of the total CoA in brain at 6 h contained PPA derived from fosmetpantotenate. Incorporation of fosmetpantotenate-derived PPA reached 50.7% at 24 h post-dose (Table 7). Mice dosed via intracerebral ventricular (ICV) cannula (1.25 and 12.5 μg) for up to 10 days showed a time- and dose-dependent increase in brain levels of +6 AMU CoA. After dosing at 12.5 μg for 10 days, these levels reached ~30% of the total CoA and were still increasing. (Table 8).

**Table 8:** Percentage of CoA derived from endogenous phosphopantothenic acid (unlabeled), fosmetpantotenate-derived pantothenic acid with rephosphorylation by WT PanK (+4 AMU), and fosmetpantotenate-derived phosphopantothenic acid (+6 AMU) following ICV administration of isotopically-labeled fosmetpantotenate to WT C57Bl6 mice^a^.

	1.25 μg ICV Brain CoA (%)			12.5 μg ICV Brain CoA (%)		
Dose Day	Unlabeled	+4 AMU	+6 AMU	Unlabeled	+4 AMU	+6 AMU
**0**	100	0	0	100	0	0
**3**	100	0	0	77	8	15
**7**	91	5	4	59	14	27
**10**	86	8	6	56	16	28

^a^ N = 6 mice per time point, pooled samples.

These data indicate that once fosmetpantotenate reaches the liver or the brain, its PPA metabolite is effectively incorporated into CoA.

## Discussion

Delivering PPA, the product of the PanK enzyme reaction, to cells has proven challenging due to its poor cell permeability [2]. To address this issue we have developed fosmetpantotenate as an enzyme product replacement for the treatment of PKAN, by designing both cell and blood-brain barrier permeability into the molecule.

First we demonstrated that fosmetpantotenate restores CoA and tubulin acetylation (a process that requires CoA). In shRNA *PANK2*-knockdown neuroblastoma cells, fosmetpantotenate increased CoA and tubulin acetylation levels. While acute doses of fosmetpantotenate at 25 to 200 μM increased free CoA levels by 2- to 4-fold, a low dose of the compound over 5 days showed little effect on free CoA, but an approximately 2.5 fold increase in total CoA. Our current hypothesis is that the initial increase in CoA upon treatment with fosmetpantotenate is quickly distributed into the many metabolic pathways that depend on CoA. Thus, we see a large increase in the ratio of total CoA to free CoA.

Tubulin acetylation, a process dependent on CoA, is also defective in our cellular model of PKAN [23]. Treatment with fosmetpantotenate restored levels of acetyl tubulin. The level of restoration was similar following 5 days of fosmetpantotenate dosing at 1 μM TID when compared to 2 days of QD dosing at 25, 50, or 200 μM. These data suggest that the effect of a low dose of fosmetpantotenate over several days is essentially equivalent to a higher dose over a shorter time period.

Another aim was to show that PPA derived from fosmetpantotenate is incorporated into CoA. This was demonstrated by dosing animals with isotopically-labeled PPA, containing tracers in the phosphate groups (^18^O) and PA groups (^15^N, ^13^C_3_). In WT mice, fully labeled (^18^O, ^15^N, ^13^C_3_)-PPA was efficiently incorporated into CoA in the liver when fosmetpantotenate was dosed orally. As fosmetpantotenate has poor stability in mouse blood, it is not surprising that, when dosed orally, no labeled CoA was seen in the brain. However, when fosmetpantotenate is delivered directly to the brain (intrastriatal or ICV administration), fully labeled (^18^O, ^15^N, ^13^C_3_)-PPA is incorporated into CoA in this tissue, indicating that once delivered to a particular site, fosmetpantotenate can serve as a precursor to CoA. In fact, up to 50% or 30% of the brain CoA in WT animals was derived from fosmetpantotenate after 125 μg intrastriatally or 12.5 μg ICV, respectively. This work confirms and expands upon a recent report from Zano and co-workers [15], who used labeled fosmetpantotenate to confirm the incorporation of PPA derived from fosmetpantotenate into CoA in the liver.

Using a 2-dimensional non-contact PBEC/rat astrocyte co-culture model adapted from the literature [17, 18], we demonstrated that fosmetpantotenate, D1, D2, and PA are permeable to the blood-brain barrier, while PPA has poor permeability. This model retains the inductive influence of astrocytes on endothelial cell barrier function and expresses key proteins associated with intercellular tight junctions and major blood-brain barrier drug transporters. Consistent with the in vitro data, fosmetpantotenate reached striatal dialysate in monkey after an oral administration.

The nature of enzyme product replacement strategies poses significant challenges with respect to demonstrating a pharmacodynamic effect and thus developing a pharmacokinetic/pharmacodynamic relationship. This is particularly acute in the case of PKAN, as it is a central nervous system disease. The rapid blood metabolism in mice and rats and the negligible exposures to fosmetpantotenate in these species were consistent with the absence of fosmetpantotenate in the brain dialysate. However, when dosed either intrastriatally or ICV, labeled CoA does form in the brain. Twenty-four hours after a single 125 μg dose into each side of the striatum, approximately 50% of the CoA is derived from labeled PPA. Furthermore, ICV administration of a 12.5 μg dose QD for 10 days results in 28% of the CoA containing the labeled atoms. These data demonstrate that once fosmetpantotenate reaches the brain, it is competent to act as a substrate in the CoA biosynthetic pathway. In non-human primates, it is significantly more difficult to demonstrate incorporation of labeled PPA derived from fosmetpantotenate because of ethical restrictions on sacrificing animals if alternative experiments are sufficient. However, we demonstrated that after both a 100 and 300 mg/kg dose of fosmetpantotenate, parent compound and several of its metabolites were found in the cerebral dialysate, indicating that in this species an oral dose leads to compound exposure in the central nervous system where it can be incorporated into CoA.

In summary, we have demonstrated that fosmetpantotenate can serve as a precursor to CoA in both in vitro and in vivo systems. This includes the IMR32 cell line with the PanK2 enzyme knocked down as well as incorporation of all labeled atoms from fosmetpantotenate when dosed to mice. Given the poor stability in mouse blood, it is not surprising that oral doses of fosmetpantotenate do not result in the appearance of labeled CoA in the brain of mice. Our data also show that the in vitro stability of fosmetpantotenate in monkeys closely mirrors that of humans. For this reason we believe that the behavior of the compound in monkeys is likely to be predictive of that in humans. In monkeys, an oral dose of fosmetpantotenate results in central nervous system exposure to the compound and several of its metabolites. These data support the continued study of fosmetpantotenate for the treatment of patients with PKAN.

## Methods

### Study facilities and approvals

All studies using animals were conducted in accordance with protocols approved by the Institutional Animal Care and Use Committee (IACUC) of the facility conducting the study, and for monkey microdialysis studies with additional approval by Niedersächsisches Landesamt für Verbraucherschutz und Lebensmittelsicherheit (Lower Saxony State Office for Consumer Protection and Food Safety, Wardenburg, Germany). Microdialysis experiments in mice were conducted at Brains On-Line (South San Francisco, CA, site). Dialysate studies in cynomolgus monkeys with blood sampling were conducted at Neu Encepharm GmbH (Gottingen, Germany), an Association for the Assessment and Accreditation of Laboratory Animal Care (AAALAC, Frederick, MD) accredited organization. Blood pharmacokinetic studies in mice, rats, and monkeys were conducted at Xenometrics LLC (Stillwell, KS), an organization accredited by AAALAC. Studies in mice to understand the fosmetpantotenate mechanism of action were conducted at IRBM in full compliance with the EU Directive 63/2010 and its Italian transposition as well as with all applicable Italian legislation and guidelines. IRBM Science Park is authorized by the Italian Ministry of Health and local veterinary authority to house, breed, and use laboratory rodents for scientific purposes. Anti-PanK2 antibodies for western blot analysis were obtained from Abcam (Cambridge, UK), catalog number 71381.

### Statistics

Statistical significance of cellular experiments was evaluated either using a two-tailed Student’s t-test as implemented in Excel within Microsoft Office 365 ProPlus or a one-way ANOVA with a Dunnett’s post-hoc analysis as implemented in GraphPad Prism version 7.00. Specific analyses are noted in the text or figure legends.

### Compound synthesis

Fosmetpantotenate was prepared in a one-pot procedure by condensation of L-alanine methyl ester hydrochloride (1.0 eq) with phenyl phosphorodichloridate (1.0 eq) in the presence of triethylamine as base and dichloromethane as solvent. Trapping of the intermediate thus formed by addition of methyl pantothenate (1.1 eq) followed by silica gel purification afforded fosmetpantotenate as an approximately 1:1 mixture of diastereomers. PA was purchased as its calcium salt (Acros, Code: 243305000, purity 98%). PPA was synthesized by palladium-catalyzed hydrogenolysis of benzyl pantothenate-4'-(dibenzyl phosphate), as previously described [24], and was stored at -20°C prior to use.

Isotopically labeled fosmetpantotenate: Isotopically labeled fosmetpantotenate was synthesized at IRBM as a 38:62 diastereomeric mixture (percentage defined by ^31^P nuclear magnetic resonance) of 97% purity, with ^18^O content of 80% (percentage defined by the isotopic pattern of the [M+Na]^+^ ion), ^13^C 99%, and ^15^N 98% (percentage established by Aldrich, the vendor, for β-alanine used for fosmetpantotenate synthesis). The ^18^O content was determined as the percent ratio of the +6 AMU signal intensity divided by the sum of the +6 and +4 AMU signal intensities, where +6 AMU and +4 AMU are the [M+Na]^+^ adducts containing (A) 3×^13^C, 1×^15^N, and 1×^18^O (^13^C_3_, ^15^N, and ^18^O); and (B) 3×^13^C, 1×^15^N, and 0×^18^O (^13^C_3_, ^15^N), respectively.

### In vitro studies

#### In vitro effect of fosmetpantotenate on coenzyme A levels following incubations in shRNA PanK2 knockdown human neuroblastoma cells

The in vitro model to evaluate the efficacy of fosmetpantotenate and analogs was developed using a human neuroblastoma IMR32 cell line (ATCC) with stable PanK2 knockdown through lentiviral-delivered shRNA [23]. This human PanK2-defective model showed a decrease of PanK2 protein levels of about 70% and 4-fold lower free and total CoA levels than control cells (data not shown), consistent with the ~2-fold decrease in CoA reported in *PanK1* KO mice when compared to WT [25]. Cells were cultured in minimal essential media (MEM; Invitrogen), supplemented with 10% fetal bovine serum, 2 mM glutamine, 1% penicillin-streptomycin, 1 mM sodium pyruvate, 1 mM non-essential amino acids, 1.5 g/L sodium bicarbonate, and 1 μg/mL puromycin. Cells were treated with 1 μM fosmetpantotenate 3 times per day (to reproduce the TID dose regimen proposed for humans) for up to 5 consecutive days at 37°C. In a separate experiment, cells were treated with 12.5, 25, 50, 100, and 200 μM fosmetpantotenate at 37°C for 24 h, followed by repeat treatment after 24 h with newly dissolved fosmetpantotenate for an additional 24 h. At the end of the time period, cells were harvested, counted, collected in a 15 mL falcon tube, and centrifuged at 200xg for 5 min at 4°C.

For all incubations, the supernatant was removed and the cell pellet was resuspended in 10 mL of ice-cold phosphate buffered saline. After centrifugation and supernatant removal, the cell pellet was extracted with 120 μL of 20% trifluoroacetic acid in water, stirred for 2 min, sonicated in an ultrasonic bath for 2 min, then stirred again for 1 min, and centrifuged for 15 min at 14000xg at 4°C. The supernatant (100 μL) was placed in a separate tube and dried under nitrogen at 20°C in the dark. Samples were reconstituted with ammonium acetate buffer (pH 5.1) containing dextrorphan (50 ng/mL) as the internal standard, stirred for 2 min, sonicated in an ultrasonic bath for 1 min, and stirred again for 1 min before liquid chromatography-tandem mass spectrometry (LC-MS/MS) analysis for determination of intracellular CoA levels. Intracellular CoA concentration was calculated considering an intracellular volume of 10^6^ cells equal to 2 μL. The measurement of the intracellular volume of cultured cells was performed following the distribution of [^14^C]urea and [^14^C]mannitol with time [26].

Coenzyme A determinations: An Agilent 1100 high-performance liquid chromatography (HPLC) system with a CTC PAL autosampler was used. Samples were chromatographed on a Phenomenex Luna C_18_ column (50 x 2.0 mm; 5 μm), with a 0.2 mL/min flow rate and a 15 μL injection volume in a 5 μL injection loop. The mobile phases were (A) 10 mM ammonium acetate in water and (B) acetonitrile:2-propanol (9:1; v:v). The initial conditions were 2% (B) maintained for 0.1 min, followed by a linear increase to reach 30% (B) at 0.12 min, and another linear increase to reach 98% (B) at 3.2 min, before returning to the starting conditions (2% [B]). A Sciex API 4000 QTrap mass spectrometer, connected to the HPLC system in tandem, with an electrospray ionization source operating in the positive scanning mode, was used for multiple reaction monitoring (MRM). Precursor ions and MRM transitions used were: for reduced CoA (CoASH), [M+H]^+^ 768.1, with transitions 768.1 to 261.1; 768.1 to 428.2; 768.1 to 136.1; for the internal standard dextrorphan, [M+H]^+^ 258.3. The statistical significance of the treatment was determined using a t-test.

#### Western blot analysis of acetylated tubulin

PanK2 knockdown cells were treated with 25, 50, and 200 μM of fosmetpantotenate as described above. After incubation, the cells were lysed in RIPA buffer containing 300 mM NaCl, 10 mM Tris HCl pH 8.0, 10 mM KCl, 1 mM ethylenediaminetetraacetic acid (EDTA), 1% Nonidet-P40, 1% sodium deoxycholate, 0.1% SDS, 1 mM phenylmethanesulfonyl fluoride, and protease inhibitor cocktail (11697498001, Roche, Indianapolis, IN), and sonicated on ice with a Branson 450 sonicator through 2 cycles of ten 2s pulses (1 min pause between cycles). Total protein concentration was determined using a Bio-Rad Protein Assay (500–0006, Biorad, Hercules, CA). Cell extracts were subjected to sodium dodecyl sulfate polyacrylamide gel electrophoresis (SDS-PAGE) analysis (4–12%, NP0335BOX, Life Technologies, Great Island, NY) and transferred to a nitrocellulose membrane (10401396, Whatman, Maidstone, UK). Immunostaining was accomplished by overnight incubation with primary antibodies followed by 1 h of incubation with dye-conjugated secondary antibodies according to the manufacturer’s instructions. Protein detection was achieved using an Infrared Odyssey system (LiCor, Lincoln, NE). Densitometric analysis of western blots was performed using ImageJ software; the statistical significance was determined using a t-test. Actin was used as a normalization probe.

#### In vitro blood-brain barrier permeability assessment

The in vitro blood-brain barrier permeability of fosmetpantotenate, its isolated diastereomers D1 and D2, PA, and PPA was assessed in vitro in a 2-dimensional non-contact model formed by PBECs in co-culture with rat astrocytes adapted from Patabendige and co-workers [17, 18].

Permeability studies were performed after 2 days of co-culture with rat astrocytes. D1, D2, PA, and PPA were stable for up to 60 min of incubation with endothelial cells. In each filter used for transport, a paracellular marker was included as an internal control to monitor the integrity of the cell monolayer. The day of the experiment, culture medium was removed and cells on Transwell inserts were pre-incubated for 30 min with Hank's Balanced Salt Solution (HBSS) containing 20 mM Hepes pH 7.4 and 0.1% bovine serum albumin (BSA). Transport via the paracellular pathway was assessed by addition of 2.5 μM fluorescein isothiocyanate (FITC)-labeled dextran (40 kDa) to the apical compartment. Aliquots from the basal compartment were taken after 1 h; fluorescence was measured using a fluorescence detector. The permeability coefficient of the total system was calculated as follows:

$$P[cm/s]=\frac{Vd\times \Delta Mr}{A\times Md\times \Delta t}$$

Where:

Vd: volume in the donor compartment in cm^3^ or mL

ΔMr: total amount of compound in the receiver compartment after t seconds

Md: donor amount (added at time 0)

Δt: time measured in seconds

A: filter area in cm^2^ (for 24-well plate A = 0.7 cm^2^)

To correct for the contribution of the porous polycarbonate membrane of the filter in the absence of cells, the permeability was also determined for the cell-free system. Contribution of the endothelial cell monolayer to the permeability of the cell and filter system was determined by the following equation:

$$\frac{1}{Pe}=\frac{1}{Pt}-\frac{1}{Pf}$$

Where the total permeability of the system is denoted as Pt, the cell-free filter as Pf, and the endothelial cell layer alone as Pe. The total resistance of the system towards passage of a substance is composed of 2 resistances in series: that of the cell monolayer and that of the filter; permeability is estimated as 1/resistance [27]. All tested compounds had a mass balance ≥80%. Mass balance was determined based on the amount of compound recovered in the donor and receiver chamber at the end of the assay relative to the amount added to the donor chamber at time 0.

#### In vitro metabolic stability

Stability in simulated gastric fluid: A stock solution of fosmetpantotenate or its individual diastereomers was prepared in 0.1% formic acid in methanol. Simulated gastric fluid, pH 1.2, was prepared with 2 g/L sodium chloride, 3.2 g/L of pepsin, and 0.7% (v/v) HCl. Gastric buffer, pH 1.2, was prepared containing 2 g/L sodium chloride and 0.7% (v/v) HCl. An appropriate aliquot of fosmetpantotenate or individual diastereomers to yield an initial concentration of 10 μM was added to both fluids, and samples were mixed at ~25°C on an orbital shaker. Aliquots (50 μL) were taken at 0, 2, and 4 h; added to 150 μL dimethyl sulfoxide (DMSO) containing an analytical internal standard; vortexed; and analyzed immediately by LC-MS/MS.

Metabolic stability in blood and liver microsomes: Fosmetpantotenate or its individual diastereomers (5 μM, n = 2, 37°C) were incubated in mouse, rat, cynomolgus monkey, and human whole blood. Samples (50 μL) were taken at 0, 5, 15, 30, and 60 min. Incubations of fosmetpantotenate or its individual diastereomers (1 μM, n = 2, 37°C) in pooled liver microsomes (0.25 mg protein/mL in 0.1 M phosphate buffer pH 7.4) were initiated with the addition of nicotinamide adenine dinucleotide phosphate (NADPH; 1 mM). Aliquots (100 μL) were obtained at 0, 5, 10, 20, and 40 min and added to 150 μL of 0.1% formic acid in acetonitrile-containing carbamazepine (0.5 μM) as an analytical internal standard, centrifuged, and the supernatants analyzed by LC-MS/MS.

Bioanalysis for determinations of analyte stability: Samples were analyzed using a benchtop tandem quadrupole detector (TQD) mass spectrometer (Waters Ltd, Centennial Park, Hertfordshire). An electrospray ionization source was used, scanning in the positive mode; the transition monitored (sodium ion) was 497.25 to 238.17. Suitable retention and peak shape with separation of the 2 diastereomers were obtained. The chromatography column was a Phenomenex-Kinetex XB C_18_ (2.10 x 50 mm; 2.6 μm), with a 0.7 mL/min flow rate and a 2 μL injection volume. Mobile phase (A) consisted of 0.1% formic acid in water; while mobile phase (B) consisted of 0.1% formic acid in acetonitrile. The gradient was initiated with 20% B and increased linearly, reaching 40% B at 0.6 min, followed by 50% B at 0.8 min and by 95% B at 0.9 min. Conditions were kept isocratic through 1.2 min, returned to starting conditions (20% B) at 1.25 min, and maintained at 20% B through 1.5 min before the next injection. Since fosmetpantotenate is a mixture of diastereomers which are resolved chromatographically, the fosmetpantotenate MRM area corresponds to the sum of the MRM areas of the individual diastereomers.

The percent of compound remaining was determined from the MRM area response in each sample relative to that in the T = 0 samples (normalized for internal standard). Natural log plots of the percent compound remaining over time were used to determine the t½ of compound disappearance using the relationship t½ = -0.693/λ, where λ is the slope of the natural logarithm of the percent compound remaining vs time curve.

### In vivo experiments

#### General animal care

Animals were housed as described below.

C57Bl6 mice were individually housed in polycarbonate cages and had access to food and water *ad libitum*. Animals were acclimated for at least 7 days prior to study start. Room temperature was maintained at 22±2°C, approximately 50% humidity, with a 12/12 hour light/dark cycle. Sawdust as bedding, Perspex red tubes, and wooden barrels were used as enrichment.

CD1 mice were housed in groups of 5 per cage in Techniplast 1248 individually vented cages, with a 12/12 hour light/dark cycle, and were acclimated for 3 days prior to study start.

Sprague-Dawley rats were housed in standard laboratory suspended cages and acclimated for a minimum of 4 days prior to the study. The room temperature was maintained at 22±4°C, with a relative humidity of 50±20%. The environment was maintained with a 12/12 light/dark cycle and 10 fresh air changes per hour. With the exception of the pre-dose fasting period, food and water were available *ad libitum*.

Monkey cages complied with European directive 2010/63/EU section B table 6.3. Adequate equipment for brachiating, climbing, and hiding and material for playing were provided. The temperature was maintained above 22°C, and the relative humidity was around 60% for the duration of the study. The animals were provided with nutritionally balanced monkey chow and mixed fresh fruits and vegetables. Animals were closely monitored throughout the study, and received treats, such as raisins and peanuts, as well as “personal contact” by personnel with whom they were familiar.

#### Pharmacokinetics in whole blood

Pharmacokinetic animal dosing and sample collection: Adult fasted male CD-1 mice (N = 4 per time point) and Sprague-Dawley rats (N = 3 per time point) from Charles River Laboratories (Kingston, NY), and *Macaca fascicularis* (cynomolgus monkeys; source, Xenometrics non-naïve colony) (N = 2 serially bled), received a single oral dose of vehicle (N = 2 per time point for vehicle) or fosmetpantotenate formulated as a solution in 20% hydroxypropyl-beta-cyclodextrin in 50 mM citric acid in water pH 5.5. Dose levels for mice and rats were 100, 300, or 700 mg/kg at 10 mL/kg and 300 mg/kg for monkeys at 5 mL/kg. Blood samples were collected via cardiac puncture for rodents pre-dose and at 0.033, 0.083, 0.167, 0.33, 0.5, 1, 2, 4, and 8 h, and via femoral vein for monkeys pre-dose and at 24 h only. The whole blood samples (0.5 mL) were collected into syringes that were prefilled with 2.5 mL of ice-cold 0.1% formic acid in acetonitrile. The volume ratio of the whole blood to 0.1% formic acid in acetonitrile was 1/5 (v/v). Samples were frozen (-70°C) until analyzed by LC-MS/MS.

Sample preparation for blood pharmacokinetic samples: Concentrations of fosmetpantotenate, PA, and PPA in whole blood were determined by Nextcea, Inc, Woburn, MA, using LC-MS/MS. Standard curves and quality control (QC) samples were prepared fresh on the day of analysis by serial dilution of the standard mixture in quenched whole blood (1 part of blood to 5 parts of 0.1% formic acid in acetonitrile) and centrifuged. Standards and QC samples contained a mixture of fosmetpantotenate and the corresponding heavy isotopes ^15^N/^13^C3-PPA (synthesized at IRBM Science Park SpA, Pomezia, RM, Italy) and ^15^N/^13^C3-PA (IsoSciences, King of Prussia, PA) as surrogates for the potential endogenous analytes PA and PPA. The frozen study samples were thawed in an ice/water bath and centrifuged. Supernatant (50 μL) from standards, QC samples, and study samples was transferred to a 96-well plate and dried completely under nitrogen. The dried residues were reconstituted with 100 μL of 0.1% formic acid in water containing internal standard (5 ng/mL of ^15^N/^13^C3-RE-024-02) and analyzed by LC-MS/MS.

LC-MS/MS analysis for blood pharmacokinetic samples: Samples were analyzed using an Agilent 1100 binary pump (Agilent Technologies, Santa Clara, CA, USA), connected to an AB Sciex API 6500 mass spectrometer (SCIEX, Framingham, MA, USA). An electrospray ionization source was used, scanning in the positive mode. The transitions monitored were as follows: fosmetpantotenate (D1 and D2) 475.1 to 216.1; PA 220.0 to 90.0; PPA: 300.3 to 202.3; ^15^N/^13^C_3_ fosmetpantotenate 479.2 to 219.9; ^15^N/^13^C_3_—PA: 224.4/94.3; and ^15^N/^13^C_3_—PPA: 304.4 to 206.3. The chromatography column was a Varian Metasil AQ 120–5 C18 column, 5 μm, 50 x 2.00 mm (Part No. A0530050X020) at ambient temperature, with a 0.5 mL/min flow rate and a 20 μL injection volume. Mobile phase (A) consisted of 0.1% formic acid in water; while mobile phase (B) consisted of 0.1% formic acid in acetonitrile:water (9:1, v/v). The gradient initiated at 0% B and increased linearly, reaching 20% B at 0.5 min, 40% B at 2.8 min, and 80% B at 4.0 min. Conditions were kept isocratic at 80% B until 4.5 min and then returned to starting conditions (0% B) over the next minute before the subsequent injection. Fosmetpantotenate is a mixture of diastereomers D1 and D2, which were resolved chromatographically, with retention times of 3.76 and 3.89 min, respectively. Retention times were 1.45 min for PPA and 1.59 min for PA.

#### Microdialysis experiments

Animal surgery: Before the microdialysis experiments, male C57Bl6 mice were housed individually in plastic cages, and monkeys were group-housed in rooms. All animals had access to food and water ad libitum and were kept on a 12/12 h light/dark cycle. Animals were acclimated for at least 5 days before surgery.

Mice were anesthetized using isoflurane (2%, 800 mL/min O_2_). Bupivacaine/epinephrine was used for local analgesia and carprofen was used for peri-/post-operative analgesia. The animals were placed in a stereotaxic frame (Kopf instruments, USA).

For oral dosing experiments, MetaQuant microdialysis probes (regenerated cellulose membrane, BrainLink, the Netherlands) were inserted into the striatum (3 mm exposed surface). Coordinates for the tips of the probes for mice were anterior-posterior (AP) = +0.8 mm from bregma, lateral (L) = -1.7 mm from midline, and ventral (V) = -4.0 mm from dura, the toothbar set at 0 mm.

For local administration (intrastriatal or unilateral ICV, mice only), a hole for the ICV cannula was drilled in the skull, taking care not to damage the underlying dura or brain tissue. Injection cannulas (Plastics One) were then inserted into the lateral ventricle. Coordinates for the tips of the cannula for the lateral ventricle were: anterior-posterior (AP) = -0.3 mm to bregma, medial-lateral (ML) = +0.9 mm from midline, and ventral (V) = -1.6 mm to dura (infusion cannula extended to -1.7 mm); the toothbar was set at 0 mm.

After surgery, animals were kept individually in cages, and provided food and water ad libitum. Microdialysis was performed on the following day.

Monkeys were sedated by intramuscular (i.m.) ketamine administration and cannulated for intravenous (i.v.) administration of propofol infusion (initiated 15–30 min after ketamine administration). Subsequently, the animals were placed in an magnetic resonance imaging (MRI)-compatible stereotaxic frame (Kopf Model 1430), and T1 and T2 weighted scans (for up to 1 h) were carried out with a 7 cm loop-coil in a 3 Tesla Siemens Magnetom TIM Trio (Germany). To derive the coordinates, Digital Imaging and Communications in Medicine (DICOM) files were transformed into Neuroimaging Informatics Technology Initiative (NIFTI) format. NIFTI-compatible images were loaded into 3D viewer software (MRIcro version 1.40 build 1), and the stereotaxic zero in the MRI image was determined using the coordinates of the earbar-tips. Coordinates for placement of the guide cannulas were calculated for every individual animal by use of MRI images and a primate brain map [28].

On a separate day, microdialysis guide cannulas were placed bilaterally in the head of the caudate nucleus using the same MRI compatible stereotaxic frame (Kopf Model 1430), under isoflurane anesthesia. Prior to surgery, animals were administered ketamine (i.m.), and subsequently anesthetized with isoflurane (0.8%-2% inhalation) while artificially ventilated with O_2_/N_2_O (40/60) at 60 to 80 mL tidal volume via an intratracheal tube. During surgery, subcutaneous or i.v. fluids were provided. Animals were allowed to recover from the anesthesia in a warmed environment, single housed, and given at a minimum a 14-day post-surgery recovery time. The following medications were administered to reduce pain and inflammation, as well as reduce the chance of post-operative infection: meloxicam (Metacam 5 mg/mL, 0.2 mg/kg, buprenorphine (Temgesic, 0.3 mg/mL, 0.0038 mg/kg), enrofloxacine (25 mg/mL, 5 mg/kg), and amoxicillin (150 mg/mL, 15 mg/kg).

Microdialysis animal dosing and sample collection: Adult non-fasted male C57Bl6 mice (20–29 g; Harlan Laboratories, Livermore, CA), and *Macaca fascicularis* (4–9 kg, 3–6 yr old) were used for the experiments. For oral dosing, fosmetpantotenate was formulated as a solution in 20% hydroxypropyl-beta-cyclodextrin in 50 mM citrate buffer in water pH 5.0 and administered using a dose volume of 10 mL/kg. Mice (N = 6 serially sampled) received fosmetpantotenate as a single oral dose at 700 mg/kg and monkeys received a single oral dose at either 100 mg/kg (N = 4 serially sampled) or 300 mg/kg (N = 2 serially sampled). In a separate experiment, mice (N = 5 serially sampled) received a direct administration at 125 μg into the striatum via an infusion cannula. Fosmetpantotenate was formulated as a 25 mg/mL solution in artificial cerebrospinal fluid (aCSF), and a dose was infused at a rate of 1.0 μL/min for 5 minutes.

Microdialysis: On the morning of the microdialysis experiment, a MetaQuant probe was inserted into the guide shaft targeting the head of the caudate nucleus and perfused at a rate of 0.15 μL/min aCSF containing 0.2% (w/v) BSA, with a carrier flow of 0.8 μL/min of ultrapure water. Microdialysis samples were collected for 30-minute periods using an automated fraction collector into low-binding microvials containing 6 μL 0.04% ascorbic acid in 0.02M (0.092%) aqueous formic acid.

A single 30 min pre-dose basal dialysate sample was collected from mice over -0.5 to 0 h, and three 30 min basal samples from monkeys over -1.5 to 0 h. Fosmetpantotenate was administered at t = 0, and 30 min dialysate samples were collected for an additional 4 h for mice and 3 h for monkeys. Samples were stored in -80°C.

From monkeys only, whole blood samples (0.5 mL) were collected from the femoral vein at -1, 0, 1, 2, and 3 h relative to dosing and transferred immediately to chilled polypropylene 15 mL vials containing 2500 μL of 0.15% formic acid in acetonitrile, mixed, and placed on dry ice. After the collections samples were stored at -80°C.

Sample preparation for mouse dialysate samples: Concentrations of fosmetpantotenate, PA, and PPA in mouse dialysate were determined by Nextcea, Inc, Woburn, MA, using LC-MS/MS. Standard curves and QC samples containing fosmetpantotenate (D1 and D2) and the surrogate analytes for PA and PPA (^15^N/^13^C3-PA and ^15^N/^13^C3-PPA) were prepared fresh in 0.1% formic acid in water. Frozen study mouse dialysate samples were thawed in ice/water bath. Standards, QC samples, and thawed study dialysate samples (20 μL) were mixed with 20 μL 0.1% formic acid in water containing internal standard (50 ng/mL of ^15^N/^13^C_3_-fosmetpantotenate) and analyzed by LC-MS/MS.

LC-MS/MS analysis for mouse dialysate samples: Concentrations of fosmetpantotenate, PA, and PPA in dialysate from mice were determined by using LC-MS/MS conditions described for blood PK analysis by Nextcea, except dialysate samples were analyzed using an ACIEX API 5000 and flow rate was increased to 0.6 mL/min.

Sample preparation for monkey dialysate and blood samples: Concentrations of fosmetpantotenate (D1 and D2), PA, and PPA in monkey dialysate and blood samples were determined by Brains On-Line LLC. On the day of analysis, standard curves and QC samples containing fosmetpantotenate (D1 and D2), PA, and PPA were prepared and study samples were thawed in ice/water bath. For dialysate quantitation, standards and QC samples were prepared in control dialysate (0.2% BSA in aCSF: water: 0.04% ascorbic acid in aqueous 0.02 M formic acid [4.5:24:6 v/v/v]). Standards, QC samples, and dialysate (10 μL) were mixed with 6 μL 20 mM formic acid and 0.01% ascorbic acid in water containing internal standard (10 ng/mL of ^15^N/^13^C_3_-PA) and analyzed by LC-MS/MS. Blood standards and QC samples were prepared in supernatant from control monkey blood which had been treated with 5 volumes of 0.15% formic acid in acetonitrile. Endogenous levels >LLOQ were detected for PA in control quenched blood only and quantitated at 56.56 nM based on calibration standard prepared in solvent. To compensate for endogenous PA in the calibration curve control blood, PA standard curve concentrations were spiked plus endogenous levels. Sample work-up of blood involved diluting 10 μL supernatant from study blood samples, standards, and QC samples with 90 μL of 20 mM formic acid and 0.01% ascorbic acid in water, then treating with 4 μL 20 mM formic acid and 0.01% ascorbic acid in water containing internal standard (10 ng/mL of ^15^N/^13^C_3_-PA) and analyzing by LC-MS/MS.

LC-MS/MS analysis for monkey dialysate and blood samples: Concentrations of fosmetpantotenate, PA, and PPA in dialysate and blood were determined using LC-MS/MS. Samples were analyzed using an Agilent 1290 binary pump (Agilent Technologies, Santa Clara, CA, USA), connected to an AB Sciex API 5500 QTRAP mass spectrometer (SCIEX, Framingham, MA, USA). An electrospray ionization source was used scanning in the positive mode for fosmetpantotenate and negative mode for PA and PPA. The transitions monitored were as follows: fosmetpantotenate (D1 and D2) 475.2 to 216.2, PA 217.6 to 88.0, PPA: 298.0 to 79.0, ^15^N/^13^C_3_—PA: 221.6 to 92.0. The chromatography column was a Waters Atlantis T3 C18, 3 μm, 150 x 2 mm at 30°C, with a 0.25 mL/min flow rate and a 20 μL injection volume. Mobile phase (A) consisted of 0.1% formic acid in water and mobile phase (B) consisted of 0.1% formic acid in acetonitrile. Conditions were kept isocratic at 5% B until 0.5 min. From 0.5 to 11.5 min the gradient increased linearly, reaching from 5% to 60% B. Conditions were kept isocratic at 95% B from 11.51 until 13.0 min then returned to starting conditions (5% B) through 15.0 min before the next injection. Fosmetpantotenate D1 and D2 diastereomers were resolved chromatographically with retention times of 10.1 and 11.1 min, respectively. Retention times were 2.60 min for PPA and 4.72 min for PA.

Quantitation of in vivo samples: Peak areas were integrated using Analyst software (AB Sciex, Concord, Ontario, Canada). Calibration curves for all blood samples from mouse and rat PK studies and mouse dialysate samples were prepared by plotting the analyte to internal standard (^15^N/^13^C3-fosmetpantotenate) peak area ratio vs concentration. Calibration curves from blood and dialysate from the monkey dialysate studies were prepared for PA by plotting the analyte to internal standard (^15^N/^13^C3-PA) peak area ratio vs concentration, and for fosmetpantotenate and PPA, by plotting the analyte peak area vs concentration. Calibration standards (at a minimum 7 per curve) were fit by linear regression with (1/x^2^) weighting. QC samples were used (triplicate) at 3 concentrations for all tandem mass spectrometry (MS/MS) runs.

All reported concentrations have been adjusted for dilution of sample during collection. The dialysis dilution correction factor was 7.66 and calculated by (total collected sample volume) / (dialysate sample volume) = (4.5 μL dialysate + 24 μL water carrier flow + 6 μL of 0.04% ascorbic acid in aqueous 0.02M formic acid)/ 4.5 μL dialysate = 34.5 mL /4.5 mL = 7.66. The blood dilution correction factor was 6 and calculated by (total sample volume) / (blood sample volume) = (0.5 mL blood + 2.5 mL 0.15% formic acid in CH3CN)/ 0.5 mL blood = 3.0 mL /0.5 mL = 6.

The reported fosmetpantotenate concentrations are the total concentrations of the individual diastereomers D1 and D2.

Pharmacokinetic calculations: PK analysis was performed using Phoenix WinNonlin^®^, Build 6.3.0.395 (Certara, Princeton, NJ, USA) using a non-compartmental model (PO dose = model 200 for extravascular dosing). The AUC was calculated using the linear trapezoidal method, where AUC_All_ = area under the concentration-time curve from time 0 to last detectable concentration, where at time 0, a concentration of zero was used for fosmetpantotenate and zero values were also used for PA or PPA if concentrations were <LLOQ. Post-dose concentrations below the limit of quantitation (BLQ) were omitted from calculation. Reported PK parameters were calculated using the mean concentration of each time point from the rat and mouse pharmacokinetics studies, and the mean of individual PK parameters for mouse dialysate and monkey PK and dialysate studies.

#### Labeled experiments

Animal phase: C57BL6N mice, 8-week old and 19 to 21 g obtained from Charles River (Como, Italy) were used in these studies. One group of mice (N = 3 per time point) received a single oral dose of 500 mg/kg isotopically labeled fosmetpantotenate dissolved in 20% hydroxypropyl-β-cyclodextrin. Another set of mice (N = 8 per time point) received a direct administration of isotopically labeled fosmetpantotenate at 125 μg (25 mg/mL of aCSF containing 147 mM NaCl, 3.0 mM KCl, 1.2 mM CaCl_2_, and 1.2 mM MgCl_2_) via cannula into striatum. Brain, liver, and blood samples were collected at pre-dose, 6 h, 12 h (brain only), and 24 h, and were snap frozen in liquid nitrogen.

Two additional groups of mice (N = 6 per time point for each group) received 3 daily doses (8 am, 2 pm, and 8 pm to mimic an anticipated human dosing schedule) of isotopically labeled fosmetpantotenate at either 1.25 μg (0.625 mg/mL aCSF) or 12.5 μg (6.25 mg/mL aCSF) via ICV infusion cannula (2 μL), for up to 10 consecutive days. Brain (striatum) samples were collected at pre-dose, and at 12 h following the third dose on days 1, 3, 7, and 10, and were snap frozen in liquid nitrogen.

Sample processing: Brain (striatum) and liver tissues were homogenized in a Precellys homogenizer (Bertin technologies, Montigny-le-Bretonneux, France) with 3 volumes of water for each volume of tissue (1:4 dilution). Protein was precipitated by adding 500 μL methanol to 100 μL of homogenate (1:6 dilution), mixed well, and centrifuged. Blood cells (1 volume) were treated with 1:8 v:v water:methanol (1:10 dilution), mixed, and centrifuged. The supernatants (200 μL for brain and blood cells and 100 μL for liver) were dried under nitrogen, reconstituted with 100 μL ammonium acetate 10 mM (pH ~5), and injected (10 μL) into the LC for high resolution mass spectrometry (HRMS) analyses.

CoA determination: An Acquity UPLC (Waters) system was used. Samples were chromatographed on a Luna C_18_ column (50 x 2.0 mm; 5 μm), with a 0.4 mL/min flow rate and a 10 μL injection volume. The mobile phases were (A), 10 mM ammonium acetate in water, pH 6.8 and (B) acetonitrile:2-propanol (9:1; v:v). The gradient was started with 2% (B) and was increased linearly to reach 20% (B) at 5 min; the gradient was ramped to reach 90% (B) at 5.1 min and kept isocratic at 90% (B) through 7.1 min. Conditions were returned to initial 2% (B) at 7.2 min, and the column was equilibrated at initial conditions until the run reached 11.2 min.

The detector was a Waters QTOF Synapt mass spectrometer, with an electrospray ion source operating in the positive scanning mode acquiring in full scan mode (accuracy <5 ppm). The following exact masses were determined: 768.1230 for CoA, 772.1301 for CoA +4 AMU, and 774.1344 for CoA +6 AMU.

The CoA levels obtained by MS/MS analyses were corrected for ^18^O isotopic incorporation. CoA levels were not adjusted for the 1% and 2% of ^12^C and ^14^N, respectively, in the labeled fosmetpantotenate. The amount of CoA formed after dosing fosmetpantotenate was calculated using the observed percent in the isotopic pattern and correcting for the ^18^O content of labeled fosmetpantotenate as follows: The % CoA derived from intact fosmetpantotenate-PPA = (100/^18^O% in prodrug) x % 6 AMU, while the % CoA derived from fosmetpantotenate-derived PA (or from oxygen exchange) = % +4 AMU—((100-^18^O% in prodrug)/^18^O% in prodrug) x % +6 AMU, where % +6 AMU and % +4 AMU are the percent of the +6 AMU and +4 AMU [M+H]^+^ CoASH molecular ions experimentally determined by HRMS from the isotopic pattern in brain, liver, and blood samples.

## Supporting information

S1 Fig (A) Target specificity of the *PANK2*_shRNA construct used for silencing. (B) Densitometry values from western blot (S1 A Fig) quantification.
(DOCX)

S2 Fig Western blot analysis from two experiments assessing the effect of three-times-daily dosing of 1 μM fosmetpantotenate over 5 days on the level of acetyl tubulin in Pank2 knockdown cells. (DOCX)

S1 Table Acetyl tubulin levels. (DOCX)

S2 Table Percent remaining of fosmetpantotenate and its individual diastereomers in human, mouse, rat, and monkey whole blood. (DOCX)

S3 Table Blood-brain barrier data. (DOCX)

S4 Table Pharmacokinetic measurements of fosmetpantotenate, PA, and PPA after oral treatment in CD-1 mice and Sprague-Dawley rats (vehicle, 100 mg/kg, 300 mg/kg/, or 700 mg/kg) and cynomolgus monkeys (300 mg/kg). (DOCX)

S5 Table Metabolite concentrations after dosing fosmetpantotenate PO in CD-1 mice, Sprague-Dawley rats, and cynomolgus monkeys. (DOCX)

S6 Table Concentrations of fosmetpantotenate, PPA, and PA in dialysates and blood after a single oral dose of fosmetpantotenate in non-fasted male C57/Bl6 mice (700 mg/kg or 125 μg) or in cynomolgus monkeys (100 or 300 mg/kg). (DOCX)

S7 Table Fig 3 (Main Manuscript) data. (DOCX)

S8 Table Fig 4 (Main Manuscript) data. (DOCX)
