# Supplementary material for: Fosmetpantotenate (RE-024), a phosphopantothenate replacement therapy for pantothenate kinase-associated neurodegeneration: Mechanism of action and efficacy in nonclinical models
Source: PLoS One. 2018 Mar 9;13(3):e0192028. doi: 10.1371/journal.pone.0192028 (PMC5844530; doi:10.1371/journal.pone.0192028)
Supplement: S1 Fig — (A) Target specificity of the PANK2_shRNA construct used for silencing. (B) Densitometry values from western blot (S1 A Fig) quantification. (DOCX) [file pone.0192028.s001.docx]

### S1 Fig Supplementary Information

Western Blot analysis using commercial antibodies previously tested for specificity in human brain lysate and purified recombinant PanK2 protein (data not shown). Protein detection was achieved by using an Infrared Odyssey system (LiCor). Densitometric analysis of western blots (S1 Fig) was performed by using ImageJ software. β-Actin was used as normalization probe for whole cell extracts. The precursor isoform of PanK2 (62.7 kDa isoform) was found to be 80% downregulated in comparison to levels in clones transfected with either empty or scramble vectors (S1 B Fig).

The human precursor form of the PANK2 enzyme (62.7 kDa) is known to undergo proteolytic cleavage during mitochondrial entry to generate a long-lived and mature isoform of about 48 kDa. Levels of the mature isoform of PanK2 were therefore also investigated in the selected PanK2 knockdown clone. Quantitative analysis revealed that in similar fashion to the precursor the levels of the mature long-lived PANK2 protein were also significantly downregulated (S1 B Fig).

**S1 Fig.**

**A**

**B**


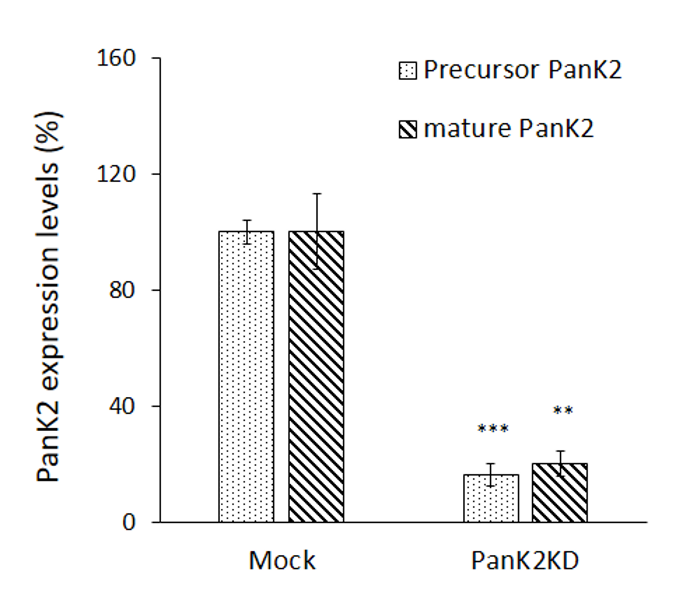


**S1 Fig**: (A) Target specificity of the *PANK2*_shRNA construct used for silencing. Western blot analysis of cell extracts (30 µg per lane) from cells transfected with the shRNA_*PANK2* (PanK2KD), with an empty control vector (Mock) using anti-PanK2 antibodies. β-Actin was used for normalization. (B) Densitometry values from western blot (S1 Fig) quantification. Data are expressed as percentage of the mock-transfected cells. ***p≤0.01, ***p≤0.001*
