# Supplementary material for: Fosmetpantotenate (RE-024), a phosphopantothenate replacement therapy for pantothenate kinase-associated neurodegeneration: Mechanism of action and efficacy in nonclinical models
Source: PLoS One. 2018 Mar 9;13(3):e0192028. doi: 10.1371/journal.pone.0192028 (PMC5844530; doi:10.1371/journal.pone.0192028)
Supplement: S2 Fig — (DOCX) [file pone.0192028.s002.docx]

**S2 Fig Supplementary Information**

**Experiment 1**

*1 µM Fosmetpantotenate*

D0

D1

D3

D5

β-actin

acetyl tubulin

37

75

50

*vehicle*

D0

D1

D3

D5

37

75

50

β-actin

acetyl tubulin

**Experiment 2**


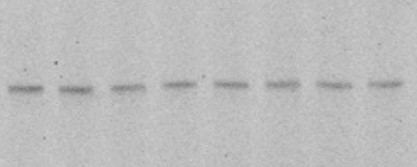

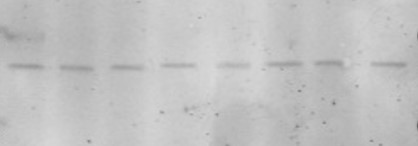


β-actin

acetyl tubulin

55-

70-

35-

55-

T0

*Vehicle (0.1% DMSO)*

D1

D3

D5


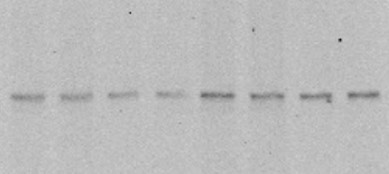

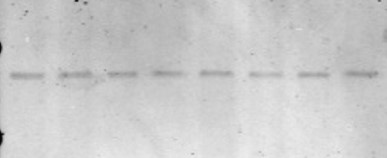


55-

70-

35-

55-

β-actin

acetyl tubulin

T0

*1 µM Fosmetpantotenate*

D1

D3

D5

**S2 Fig:** Western blot analysis from two experiments assessing the effect of TID dosing of 1 µM fosmetpantotenate over 5 days on the level of acetyl tubulin in Pank2 knockdown cells. Experiment 1 – 25 µg of protein loaded, anti-α-acetyl tubulin antibody (T6793, Sigma) 1:5000, anti-actin antibody (A2066, Sigma) 1:5000; Experiment 2 – 10 µg of protein loaded, anti-α-acetyl tubulin antibody (T6793, Sigma) 1:2500, anti-actin antibody (A2066, Sigma) 1:5000
