# Supplementary material for: Fosmetpantotenate (RE-024), a phosphopantothenate replacement therapy for pantothenate kinase-associated neurodegeneration: Mechanism of action and efficacy in nonclinical models
Source: PLoS One. 2018 Mar 9;13(3):e0192028. doi: 10.1371/journal.pone.0192028 (PMC5844530; doi:10.1371/journal.pone.0192028)
Supplement: S1 Table — (DOCX) [file pone.0192028.s003.docx]

**S1 Table. Acetyl tubulin levels.**

|  | Acetyl tubulin level (fold over vehicle) | | | |
| --- | --- | --- | --- | --- |
|  | Exp1 (n=1) | Exp2 (n=3) | Exp3 (n=3) | Mean Exp1, 2, 3 |
| PanK2 KD vehicle | 1 | 1 ± 0.04 | 1 ± 0.11 | 1 |
| RE-024 25µM | 1.86 | n.d. | 2.77 ± 0.13 | 2.32 ± 0.64 |
| RE-024 50µM | 3.02 | 3.03 ± 0.05 | 4.99 ± 0.03 | 3.68 ± 1.13 |
| RE-024 200µM | 3.89 | 3.40 ± 0.08 | 8.08 ± 0.07 | 5.12 ± 2.57 |

RE-024: fosmetpantotenate

Experiment 1

Experiment 2

Experiment 3

RE-024: fosmetpantotenate
