# Supplementary material for: Fosmetpantotenate (RE-024), a phosphopantothenate replacement therapy for pantothenate kinase-associated neurodegeneration: Mechanism of action and efficacy in nonclinical models
Source: PLoS One. 2018 Mar 9;13(3):e0192028. doi: 10.1371/journal.pone.0192028 (PMC5844530; doi:10.1371/journal.pone.0192028)
Supplement: S2 Table — (DOCX) [file pone.0192028.s004.docx]

**S2 Table. Percent remaining of fosmetpantotenate and its individual diastereomers in human, mouse, rat, and monkey whole blood.**

**Percent remaining of fosmetpantotenate and its individual diastereomers in human whole blood**

|  |  | Normalized peak height | | Percentage Remaining | | Ln Percentage Remaining | |
| --- | --- | --- | --- | --- | --- | --- | --- |
|  | Time (min) | 1 | 2 | 1 | 2 | 1 | 2 |
| Diastereomer 1 | 0 | 49.5743 | 44.3241 | 100 | 100 | 4.605 | 4.605 |
|  | 5 | 47.5222 | 47.3250 | 96 | 107 | 4.563 | 4.671 |
|  | 15 | 43.3941 | 44.5497 | 88 | 101 | 4.472 | 4.610 |
|  | 30 | 30.3606 | 34.3899 | 61 | 78 | 4.115 | 4.351 |
|  | 60 | 18.2782 | 19.8854 | 37 | 45 | 3.607 | 3.804 |
| Diastereomer 2 | 0 | 71.2613 | 75.1629 | 100 | 100 | 4.605 | 4.605 |
|  | 5 | 80.4260 | 88.3475 | 113 | 118 | 4.726 | 4.767 |
|  | 15 | 86.4442 | 95.7155 | 121 | 127 | 4.798 | 4.847 |
|  | 30 | 60.3990 | 70.0315 | 85 | 93 | 4.440 | 4.534 |
|  | 60 | 60.9610 | 54.8222 | 86 | 73 | 4.449 | 4.290 |
| Fosmetpantotenate Total | 0 | 54.5763 | 63.3246 | 100 | 100 | 4.605 | 4.605 |
|  | 5 | 56.1797 | 56.8627 | 103 | 90 | 4.634 | 4.498 |
|  | 15 | 62.0925 | 62.6797 | 114 | 99 | 4.734 | 4.595 |
|  | 30 | 41.9422 | 45.1533 | 77 | 71 | 4.342 | 4.267 |
|  | 60 | 30.8337 | 35.0688 | 56 | 55 | 4.034 | 4.014 |

**Percent remaining of fosmetpantotenate and its individual diastereomers in mouse whole blood**

|  |  | Normalized peak height | | Percentage Remaining | | Ln Percentage Remaining | |
| --- | --- | --- | --- | --- | --- | --- | --- |
|  | Time (min) | 1 | 2 | 1 | 2 | 1 | 2 |
| Fosmetpantotenate Total | 0 | 1.0672 | 1.0672 | 100 | 100 | 4.605 | 4.605 |
|  | 5 | 0.0139 | 0.0149 | 1 | 1 | 0.266 | 0.331 |
|  | 15 | 0.0132 | 0.0139 | 1 | 1 | 0.215 | 0.261 |
|  | 30 | NA | NA | NA | NA | NA | NA |
|  | 60 | NA | NA | NA | NA | NA | NA |
|  | 90 | NA | NA | NA | NA | NA | NA |
|  | 120 | NA | NA | NA | NA | NA | NA |
| Diastereomer 1 | 0 | 0.6213 | 0.6213 | 100 | 100 | 4.605 | 4.605 |
|  | 5 | 0.0006 | 0.0030 | 0 | 0 | -2.399 | -0.723 |
|  | 15 | 0.0016 | 0.0020 | 0 | 0 | -1.334 | -1.124 |
|  | 30 | NA | NA | NA | NA | NA | NA |
|  | 60 | NA | NA | NA | NA | NA | NA |
|  | 90 | NA | NA | NA | NA | NA | NA |
|  | 120 | NA | NA | NA | NA | NA | NA |
| Diastereomer 2 | 0 | 1.3168 | 1.3168 | 100 | 100 | 4.605 | 4.605 |
|  | 5 | 0.0103 | 0.0113 | 1 | 1 | -0.244 | -0.150 |
|  | 15 | 0.0115 | 0.0127 | 1 | 1 | -0.137 | -0.040 |
|  | 30 | NA | NA | NA | NA | NA | NA |
|  | 60 | NA | NA | NA | NA | NA | NA |
|  | 90 | NA | NA | NA | NA | NA | NA |
|  | 120 | NA | NA | NA | NA | NA | NA |

**Percent remaining of fosmetpantotenate and its individual diastereomers in rat whole blood**

|  |  | Normalized peak height | | Percentage Remaining | | Ln Percentage Remaining | |
| --- | --- | --- | --- | --- | --- | --- | --- |
|  | Time (min) | 1 | 2 | 1 | 2 | 1 | 2 |
| Fosmetpantotenate Total | 0 | 118.5386 | 118.5386 | 100 | 100 | 4.605 | 4.605 |
|  | 5 | 7.0656 | 0.7709 | 6 | 1 | 1.785 | -0.430 |
|  | 15 | 0.1655 | 9.7838 | 0 | 8 | -1.969 | 2.111 |
|  | 30 | 0.1064 | 0.0918 | 0 | 0 | -2.411 | -2.558 |
|  | 60 | 0.4351 | 0.2831 | 0 | 0 | -1.002 | -1.432 |
| Diastereomer 1 | 0 | 72.9780 | 72.9780 | 100 | 100 | 4.605 | 4.605 |
|  | 5 | 7.0241 | 3.6323 | 10 | 5 | 2.264 | 1.605 |
|  | 15 | 0.1414 | 1.4572 | 0 | 2 | -1.641 | 0.692 |
|  | 30 | 0.2448 | 0.0164 | 0 | 0 | -1.092 | -3.793 |
|  | 60 | 0.1350 | 0.7830 | 0 | 1 | -1.687 | 0.070 |
| Diastereomer 2 | 0 | 119.4069 | 119.4069 | 100 | 100 | 4.605 | 4.605 |
|  | 5 | 8.2940 | 0.5496 | 7 | 0 | 1.938 | -0.776 |
|  | 15 | 0.0449 | 15.4722 | 0 | 13 | -3.280 | 2.562 |
|  | 30 | 0.0418 | 0.1538 | 0 | 0 | -3.352 | -2.049 |
|  | 60 | 0.3845 | 0.4524 | 0 | 0 | -1.133 | -0.971 |

**Percent remaining of fosmetpantotenate and its individual diastereomers in monkey whole blood**

|  |  | Normalized peak height | | Percentage Remaining | | Ln Percentage Remaining | |
| --- | --- | --- | --- | --- | --- | --- | --- |
|  | Time (min) | 1 | 2 | 1 | 2 | 1 | 2 |
| Diastereomer 1 | 0 | 0.1645 | 0.2258 | 100 | 100 | 4.605 | 4.605 |
|  | 5 | 0.1443 | 0.1995 | 88 | 88 | 4.474 | 4.481 |
|  | 15 | 0.1188 | 0.1728 | 72 | 76 | 4.280 | 4.337 |
|  | 30 | 0.1159 | 0.1486 | 70 | 66 | 4.255 | 4.187 |
|  | 60 | 0.0560 | 0.0730 | 34 | 32 | 3.528 | 3.476 |
|  | 90 | 0.0342 | 0.0417 | 21 | 18 | 3.035 | 2.915 |
|  | 120 | 0.0060 | 0.0332 | 4 | 15 | 1.296 | 2.689 |
| Diastereomer 2 | 0 | 0.2563 | 0.2991 | 100 | 100 | 4.605 | 4.605 |
|  | 5 | 0.1953 | 0.2532 | 76 | 85 | 4.333 | 4.439 |
|  | 15 | 0.1031 | 0.1253 | 40 | 42 | 3.695 | 3.735 |
|  | 30 | 0.0621 | 0.0827 | 24 | 28 | 3.187 | 3.319 |
|  | 60 | 0.0040 | 0.0133 | 2 | 4 | 0.455 | 1.489 |
|  | 90 | 0.0036 | 0.0062 | 1 | 2 | 0.333 | 0.731 |
|  | 120 | 0.0032 | 0.0032 | 1 | 1 | 0.232 | 0.083 |
| Fosmetpantotenate Total | 0 | 0.2025 | 0.1570 | 100 | 100 | 4.605 | 4.605 |
|  | 5 | 0.1597 | 0.1702 | 79 | 108 | 4.368 | 4.686 |
|  | 15 | 0.1342 | 0.1537 | 66 | 98 | 4.194 | 4.584 |
|  | 30 | 0.0914 | 0.1239 | 45 | 79 | 3.809 | 4.369 |
|  | 60 | 0.0445 | 0.0548 | 22 | 35 | 3.090 | 3.554 |
|  | 90 | 0.0309 | 0.0557 | 15 | 35 | 2.726 | 3.569 |
|  | 120 | 0.0195 | 0.0269 | 10 | 17 | 2.265 | 2.843 |
