# Supplementary material for: Fosmetpantotenate (RE-024), a phosphopantothenate replacement therapy for pantothenate kinase-associated neurodegeneration: Mechanism of action and efficacy in nonclinical models
Source: PLoS One. 2018 Mar 9;13(3):e0192028. doi: 10.1371/journal.pone.0192028 (PMC5844530; doi:10.1371/journal.pone.0192028)
Supplement: S3 Table — (DOCX) [file pone.0192028.s005.docx]

**S3 Table. Blood-brain barrier data.**

|  | **P_app_ (10^-6^ cm/s)** | | |
| --- | --- | --- | --- |
| Time (min) | **Fosmetpantotenate D1** | | |
| 60 | 2.7 | 5.5 | 6.2 |
| TEER | 578 | 574 | 507 |

|  | **P_app_ (10^-6^ cm/s)** | | |
| --- | --- | --- | --- |
| Time (min) | **Fosmetpantotenate D2** | | |
| 60 | 2.7 | 3.9 | 5.4 |
| TEER | 578 | 574 | 507 |

|  | **P_app_ (10^-6^ cm/s)** | | |
| --- | --- | --- | --- |
| Time (min) | **PA** | | |
| 60 | 5.6 | 3.7 | 3.2 |
| TEER | 758 | 819 | 660 |

|  | **P_app_ (10^-6^ cm/s)** | | |
| --- | --- | --- | --- |
| Time (min) | **PPA** | | |
| 60 | 1.2 | 0.8 | LOQ* |
| TEER | 907 | 991 | 918 |

* Excluded from calculations

LOQ: limit of quantitation; PA: pantothenate; P_app_: apparent permeability; PPA: phosphopantothenate; TEER: transepithelial electrical resistance
