# Supplementary material for: Fosmetpantotenate (RE-024), a phosphopantothenate replacement therapy for pantothenate kinase-associated neurodegeneration: Mechanism of action and efficacy in nonclinical models
Source: PLoS One. 2018 Mar 9;13(3):e0192028. doi: 10.1371/journal.pone.0192028 (PMC5844530; doi:10.1371/journal.pone.0192028)
Supplement: S4 Table — (DOCX) [file pone.0192028.s006.docx]

**S4 Table.** **Pharmacokinetic measurements of fosmetpantotenate, PA, and PPA after oral treatment in CD-1 mice and Sprague-Dawley rats (vehicle, 100 mg/kg, 300 mg/kg/, or 700 mg/kg) and cynomolgus monkeys (300 mg/kg).**

| **Pharmacokinetic measurements in CD-1 mice**  **Blood concentrations and PK parameters of fosmetpantotenate, PPA, and PA after oral treatment with vehicle in male CD-1 mice**  **Vehicle: 10 mL/kg of 20% hydroxypropyl-β-cyclodextrin (HPβCD) in citric acid (pH 5.5)** | | | | | | | |
| --- | --- | --- | --- | --- | --- | --- | --- |
| Collection Time (hr) | Analytes | Fosmetpantotenate | | PPA | | PA | |
|  | Animal No. | Conc. (nM) | Mean Conc. (nM) | Conc. (nM) | Mean Conc. (nM) | Conc. (nM) | Mean Conc. (nM) |
| 0.033 | 151 | BQL | BQL | BQL | BQL | 383.10 | 381.80 |
|  | 152 | BQL |  | BQL |  | 380.40 |  |
| 0.083 | 153 | BQL | BQL | BQL | BQL | 333.90 | 362.60 |
|  | 154 | BQL |  | BQL |  | 391.40 |  |
| 0.167 | 155 | BQL | BQL | BQL | BQL | 443.30 | 324.80 |
|  | 156 | BQL |  | BQL |  | 206.20 |  |
| 0.333 | 157 | BQL | BQL | BQL | BQL | 322.90 | 293.70 |
|  | 158 | BQL |  | BQL |  | 264.60 |  |
| 0.5 | 159 | BQL | BQL | BQL | BQL | 212.60 | 205.30 |
|  | 160 | BQL |  | BQL |  | 198.00 |  |
| 1 | 161 | BQL | BQL | BQL | BQL | 245.40 | 269.10 |
|  | 162 | BQL |  | BQL |  | 292.80 |  |
| 2 | 163 | BQL | BQL | BQL | BQL | 177.00 | 259.50 |
|  | 164 | BQL |  | BQL |  | 342.10 |  |
| 4 | 165 | BQL | BQL | BQL | BQL | 243.10 | 254.10 |
|  | 166 | BQL |  | BQL |  | 265.00 |  |
| 8 | 167 | BQL | BQL | BQL | BQL | 473.50 | 356.00 |
|  | 168 | BQL |  | BQL |  | 238.60 |  |

BQL: fosmetpantotenate <1.02 nM, PPA <20.1 nM, PA <27.4 nM

| **Blood Concentrations and PK parameters of fosmetpantotenate, PPA, and PA after oral treatment with fosmetpantotenate at  100 mg/kg in male CD-1 mice** | | | | | | | | | | |  |
| --- | --- | --- | --- | --- | --- | --- | --- | --- | --- | --- | --- |
| Collection Time (hr) | Analytes | Fosmetpantotenate | | | PPA | | | PA | | |  |
|  | Animal No. | Conc. (nM) | Mean Conc. (nM) | SD | Conc. (nM) | Mean Conc. (nM) | SD | Conc. (nM) | Mean Conc. (nM) | SD |  |
| 0.033 | 35 | 6.22 | 7.81 | ND | 437.10 | 522.10 | 479.30 | 314.70 | 318.10 | 80.70 |  |
|  | 36 | BQL |  |  | 197.50 |  |  | 377.70 |  |  |  |
|  | 37 | 218.78 ^(a)^ |  |  | 1223.20 |  |  | 374.90 |  |  |  |
|  | 38 | 9.40 |  |  | 230.60 |  |  | 205.30 |  |  |  |
| 0.083 | 39 | 1.94 | 1.97 | ND | BQL | 123.50 | 96.50 | 263.80 | 286.10 | 52.80 |  |
|  | 40 | 2.00 |  |  | 113.30 |  |  | 342.10 |  |  |  |
|  | 41 | BQL |  |  | 224.60 |  |  | 314.70 |  |  |  |
|  | 42 | BQL |  |  | 32.50 |  |  | 223.60 |  |  |  |
| 0.167 | 43 | 1.31 | 1.80 | ND | 168.80 | 326.10 | 307.00 | 229.60 | 290.10 | 59.90 |  |
|  | 44 | BQL |  |  | 114.90 |  |  | 263.00 |  |  |  |
|  | 45 | 2.30 |  |  | 780.00 |  |  | 369.50 |  |  |  |
|  | 46 | BQL |  |  | 240.60 |  |  | 298.30 |  |  |  |
| 0.333 | 47 | 1.31 | 1.74 | 0.53 | 181.90 | 188.80 | 74.30 | 241.10 | 322.30 | 60.30 |  |
|  | 48 | 1.31 |  |  | 99.10 |  |  | 369.50 |  |  |  |
|  | 49 | 2.38 |  |  | 280.70 |  |  | 312.00 |  |  |  |
|  | 50 | 1.98 |  |  | 193.70 |  |  | 366.70 |  |  |  |
| 0.5 | 51 | BQL | 1.39 | ND | 47.50 | 114.10 | 48.50 | 173.00 | 278.20 | 96.10 |  |
|  | 52 | BQL |  |  | 112.10 |  |  | 236.50 |  |  |  |
|  | 53 | 1.35 |  |  | 136.80 |  |  | 306.50 |  |  |  |
|  | 54 | 1.43 |  |  | 160.00 |  |  | 396.80 |  |  |  |
| 1 | 55 | BQL | 1.52 | ND | 49.70 | 71.10 | 52.00 | 478.90 | 472.80 | 188.00 |  |
|  | 56 | BQL |  |  | 43.10 |  |  | 292.80 |  |  |  |
|  | 57 | 1.26 |  |  | 42.50 |  |  | 388.60 |  |  |  |
|  | 58 | 1.77 |  |  | 149.00 |  |  | 730.70 |  |  |  |
| 2 | 59 | 2.97 | 2.97 | ND | 29.50 | 28.60 | 5.60 | 377.70 | 429.70 | 122.90 |  |
|  | 60 | BQL |  |  | BQL |  |  | 281.90 |  |  |  |
|  | 61 | BQL |  |  | 22.70 |  |  | 550.10 |  |  |  |
|  | 62 | BQL |  |  | 33.70 |  |  | 509.00 |  |  |  |
| 4 | 63 | BQL | BQL | ND | 21.50 | 24.10 | 2.70 | 1108.40 | 771.80 | 233.60 |  |
|  | 64 | BQL |  |  | 24.10 |  |  | 629.40 |  |  |  |
|  | 65 | BQL |  |  | 26.90 |  |  | 749.90 |  |  |  |
|  | 66 | BQL |  |  | BQL |  |  | 599.30 |  |  |  |
| 8 | 67 | BQL | BQL | ND | BQL | BQL | ND | 892.20 | 1064.60 | 374.40 |  |
|  | 68 | BQL |  |  | BQL |  |  | 823.80 |  |  |  |
|  | 69 | BQL |  |  | BQL |  |  | 1622.90 |  |  |  |
|  | 70 | BQL |  |  | BQL |  |  | 919.50 |  |  |  |
| (a) Concentration was inconsistent with cohort and not included in the mean concentration calculation. | | | | | | | | | | | |
| (b) PA PK parameters adjusted for basal levels=(PK value in fosmetpantotenate-dosed group) - (basal PA PK from control [vehicle]-dosed group). Basal C_max_=382 nM, and basal AUC=2260 nM*hr. | | | | | | | | | | | |
| BQL: fosmetpantotenate <1.02 nM, PPA <20.1 nM, PA <27.4 nM | | | | | | | | | | | |
|  | | | | | | | | | | | |
|  | | | | | | | | | | | |

| **Blood Concentrations and PK parameters of fosmetpantotenate, PPA, and PA after oral treatment with fosmetpantotenate at  300 mg/kg in male CD-1 mice** | | | | | | | | | | |  |
| --- | --- | --- | --- | --- | --- | --- | --- | --- | --- | --- | --- |
| Collection Time (hr) | Analytes | Fosmetpantotenate | | | PPA | | | PA | | |  |
|  | Animal No. | Conc. (nM) | Mean Conc. (nM) | SD | Conc. (nM) | Mean Conc. (nM) | SD | Conc. (nM) | Mean Conc. (nM) | SD |  |
| 0.033 | 75 | 1.41 | 30.19 | 33.17 | 140.60 | 2015.30 | 1995.40 | 295.60 | 475.50 | 169.20 |  |
|  | 76 | 49.95 |  |  | 3789.90 |  |  | 624.00 |  |  |  |
|  | 77 | 66.65 |  |  | 3689.60 |  |  | 615.80 |  |  |  |
|  | 78 | 2.76 |  |  | 441.10 |  |  | 366.70 |  |  |  |
| 0.083 | 79 | 28.96 | 14.44 | 11.63 | 256.70 | 187.30 | 111.60 | 394.10 | 274.50 | 110.60 |  |
|  | 80 | 5.50 |  |  | 58.60 |  |  | 342.10 |  |  |  |
|  | 81 | 4.59 |  |  | 246.60 |  |  | 193.20 |  |  |  |
|  | 82 | 18.72 |  |  | BQL |  |  | 168.60 |  |  |  |
| 0.167 | 83 | 1.62 | 2.30 | 1.22 | 1437.70 | 2586.70 | 1768.60 | 574.70 | 625.30 | 106.20 |  |
|  | 84 | 1.56 |  |  | 3749.70 |  |  | 634.90 |  |  |  |
|  | 85 | 3.71 |  |  | 4411.50 |  |  | 769.00 |  |  |  |
|  | 86 | BQL |  |  | 747.90 |  |  | 522.70 |  |  |  |
| 0.333 | 87 | 1.43 | 1.22 | 0.16 | 1108.90 | 968.00 | 154.30 | 719.80 | 602.10 | 105.20 |  |
|  | 88 | 1.22 |  |  | 1048.70 |  |  | 640.40 |  |  |  |
|  | 89 | 1.07 |  |  | 958.50 |  |  | 577.40 |  |  |  |
|  | 90 | 1.14 |  |  | 756.00 |  |  | 470.70 |  |  |  |
| 0.5 | 91 | 1.83 | 1.36 | 0.32 | 703.80 | 820.60 | 110.50 | 788.20 | 800.50 | 151.60 |  |
|  | 92 | 1.14 |  |  | 776.00 |  |  | 897.60 |  |  |  |
|  | 93 | 1.24 |  |  | 838.20 |  |  | 591.10 |  |  |  |
|  | 94 | 1.24 |  |  | 964.50 |  |  | 925.00 |  |  |  |
| 1 | 95 | BQL | 1.14 | ND | 258.70 | 185.10 | 74.60 | 1037.20 | 897.60 | 298.60 |  |
|  | 96 | BQL |  |  | 236.60 |  |  | 1245.20 |  |  |  |
|  | 97 | 1.14 |  |  | 142.60 |  |  | 588.40 |  |  |  |
|  | 98 | BQL |  |  | 102.50 |  |  | 719.80 |  |  |  |
| 2 | 99 | BQL | BQL | ND | 60.20 | 68.10 | 11.50 | 440.60 | 856.60 | 302.50 |  |
|  | 100 | BQL |  |  | 84.20 |  |  | 1042.70 |  |  |  |
|  | 101 | BQL |  |  | 59.60 |  |  | 1113.80 |  |  |  |
|  | 102 | BQL |  |  | 68.60 |  |  | 829.20 |  |  |  |
| 4 | 103 | BQL | 1.03 | ND | 74.00 | 59.10 | 14.50 | 1217.80 | 1152.80 | 277.60 |  |
|  | 104 | BQL |  |  | 64.40 |  |  | 1436.80 |  |  |  |
|  | 105 | 1.03 |  |  | 58.20 |  |  | 771.80 |  |  |  |
|  | 106 | BQL |  |  | 39.70 |  |  | 1185.00 |  |  |  |
| 8 | 107 | BQL | BQL | ND | 33.90 | 27.60 | 5.20 | 1466.90 | 1059.80 | 407.90 |  |
|  | 108 | BQL |  |  | 22.70 |  |  | 1354.70 |  |  |  |
|  | 109 | BQL |  |  | 29.90 |  |  | 711.50 |  |  |  |
|  | 110 | BQL |  |  | 24.10 |  |  | 706.10 |  |  |  |
| (a) PA PK parameters adjusted for basal levels=(PK value in fosmetpantotenate-dosed group) - (basal PA PK from control [vehicle]-dosed group). Basal C_max_=382 nM, and basal AUC=2260 nM*hr. | | | | | | | | | | | |
| BQL: below quantitation limit: fosmetpantotenate <1.02 nM, PPA <20.1 nM, PA <27.4 nM | | | | | | | | | | | |
|  | | | | | | | | | | | |
|  | | | | | | | | | | | |

| **Blood Concentrations and PK parameters of fosmetpantotenate, PPA, and PA after oral treatment with fosmetpantotenate at  700 mg/kg in male CD-1 mice** | | | | | | | | | | |  |
| --- | --- | --- | --- | --- | --- | --- | --- | --- | --- | --- | --- |
| Collection Time (hr) | Analytes | Fosmetpantotenate | | | PPA | | | PA | | |  |
|  | Animal No. | Conc. (nM) | Mean Conc. (nM) | SD | Conc. (nM) | Mean Conc. (nM) | SD | Conc. (nM) | Mean Conc. (nM) | SD |  |
| 0.033 | 115 | 61.71 | 134.67 | 91.43 | 1066.80 | 2387.20 | 1084.50 | 347.60 | 481.70 | 124.70 |  |
|  | 116 | 230.17 |  |  | 2606.80 |  |  | 476.20 |  |  |  |
|  | 117 | 51.34 |  |  | 2185.70 |  |  | 454.30 |  |  |  |
|  | 118 | 195.45 |  |  | 3689.60 |  |  | 648.60 |  |  |  |
| 0.083 | 119 | 16.06 | 16.21 | 8.00 | 4311.20 | 2725.10 | 1807.10 | 840.20 | 619.90 | 169.40 |  |
|  | 120 | 24.28 |  |  | 493.30 |  |  | 457.00 |  |  |  |
|  | 121 | 3756.01(a) |  |  | 4070.60 |  |  | 659.60 |  |  |  |
|  | 122 | 8.28 |  |  | 2025.30 |  |  | 522.70 |  |  |  |
| 0.167 | 123 | 575.42 | 307.15 | 269.00 | 8221.40 | 7589.70 | 1934.60 | 1105.60 | 1186.40 | 316.90 |  |
|  | 124 | 158.08 |  |  | 7178.70 |  |  | 957.90 |  |  |  |
|  | 125 | 6.93 |  |  | 5173.50 |  |  | 1029.00 |  |  |  |
|  | 126 | 488.15 |  |  | 9785.40 |  |  | 1653.00 |  |  |  |
| 0.333 | 127 | 24.41 | 23.59 | ND | 2426.30 | 2747.10 | 453.70 | 769.00 | 1007.10 | ND |  |
|  | 128 | 22.76 |  |  | 3068.00 |  |  | 1245.20 |  |  |  |
|  | 129 | NS |  |  | NS |  |  | NS |  |  |  |
|  | 130 | NS |  |  | NS |  |  | NS |  |  |  |
| 0.5 | 131 | 3.33 | 78.71 | 136.62 | 1415.70 | 1113.90 | 202.40 | 741.70 | 841.50 | 165.70 |  |
|  | 132 | 21.75 |  |  | 1022.70 |  |  | 840.20 |  |  |  |
|  | 133 | 6.47 |  |  | 1034.70 |  |  | 708.80 |  |  |  |
|  | 134 | 283.28 |  |  | 982.60 |  |  | 1075.50 |  |  |  |
| 1 | 135 | 128.99 | 51.57 | 59.49 | 649.70 | 384.80 | 193.30 | 1157.60 | 1020.10 | 308.80 |  |
|  | 136 | 6.41 |  |  | 322.80 |  |  | 886.70 |  |  |  |
|  | 137 | 3.33 |  |  | 377.00 |  |  | 1371.10 |  |  |  |
|  | 138 | 67.53 |  |  | 189.70 |  |  | 665.00 |  |  |  |
| 2 | 139 | 1.29 | 5.11 | 7.47 | 142.40 | 262.70 | 80.70 | 1565.40 | 1493.60 | 173.50 |  |
|  | 140 | 1.18 |  |  | 300.80 |  |  | 1338.30 |  |  |  |
|  | 141 | 1.64 |  |  | 292.80 |  |  | 1365.60 |  |  |  |
|  | 142 | 16.31 |  |  | 314.80 |  |  | 1705.00 |  |  |  |
| 4 | 143 | BQL | 1.14 | ND | 141.40 | 128.00 | 28.90 | 1981.40 | 1991.70 | 444.40 |  |
|  | 144 | BQL |  |  | 146.00 |  |  | 2452.10 |  |  |  |
|  | 145 | BQL |  |  | 84.80 |  |  | 1393.00 |  |  |  |
|  | 146 | 1.14 |  |  | 139.80 |  |  | 2140.10 |  |  |  |
| 8 | 147 | BQL | BQL | ND | 104.70 | 63.50 | 29.70 | 2900.90 | 1558.60 | 914.10 |  |
|  | 148 | BQL |  |  | 49.30 |  |  | 1349.20 |  |  |  |
|  | 149 | BQL |  |  | 36.10 |  |  | 894.90 |  |  |  |
|  | 150 | BQL |  |  | 64.00 |  |  | 1089.20 |  |  |  |
| (a) Concentration was inconsistent with cohort and not included in the mean concentration calculation. | | | | | | | | | | | |
| (b) PA PK parameters adjusted for basal levels=(PK value in fosmetpantotenate-dosed group) - (basal PA PK from control [vehicle]-dosed group). Basal C_max_=382 nM, and basal AUC=2260 nM*hr. | | | | | | | | | | | |
| BQL: below quantitation limit: fosmetpantotenate <1.02 nM, PPA <20.1 nM, PA <27.4 nM | | | | | | | | | | | |
|  | | | | | | | | | | | |

**Pharmacokinetic measurements in Sprague-Dawley Rats**

| **Blood concentrations and PK parameters of fosmetpantotenate, PPA, and PA after oral treatment with vehicle in male Sprague-Dawley Rats**  **Vehicle: 10 mL/kg of 20% hydroxypropyl-β-cyclodextrin (HPβCD) in citric acid (pH 5.5)** | | | | | | | |
| --- | --- | --- | --- | --- | --- | --- | --- |
| Collection Time (hr) | Analytes | Fosmetpantotenate | | PPA | | PA | |
|  | Animal No. | Conc. (nM) | Mean Conc. (nM) | Conc. (nM) | Mean Conc. (nM) | Conc. (nM) | Mean Conc. (nM) |
| 0.033 | 121 | BQL | BQL | BQL | BQL | 1020.80 | 1098.80 |
|  | 122 | BQL |  | BQL |  | 1176.79 |  |
| 0.083 | 123 | BQL | BQL | BQL | BQL | 1921.18 | 1717.30 |
|  | 124 | BQL |  | BQL |  | 1513.41 |  |
| 0.167 | 125 | BQL | BQL | BQL | BQL | 1505.20 | 1194.58 |
|  | 126 | BQL |  | BQL |  | 883.96 |  |
| 0.333 | 127 | BQL | BQL | BQL | BQL | 1729.61 | 1507.94 |
|  | 128 | BQL |  | BQL |  | 1286.26 |  |
| 0.5 | 129 | BQL | BQL | BQL | BQL | 1420.36 | 1579.09 |
|  | 130 | BQL |  | BQL |  | 1737.82 |  |
| 1 | 131 | BQL | BQL | BQL | BQL | 1902.03 | 1691.30 |
|  | 132 | BQL |  | BQL |  | 1480.57 |  |
| 2 | 133 | BQL | BQL | BQL | BQL | 2660.10 | 2275.59 |
|  | 134 | BQL |  | BQL |  | 1891.08 |  |
| 4 | 135 | BQL | BQL | BQL | BQL | 1937.60 | 1599.62 |
|  | 136 | BQL |  | BQL |  | 1261.63 |  |
| 8 | 137 | BQL | BQL | BQL | BQL | 2022.44 | 1876.03 |
|  | 138 | BQL |  | BQL |  | 1729.61 |  |

| **Blood concentrations and PK parameters of Fosmetpantotenate, PPA, and PA after oral treatment with fosmetpantotenate at  100 mg/kg in male Sprague-Dawley Rats** | | | | | | | | | | | |
| --- | --- | --- | --- | --- | --- | --- | --- | --- | --- | --- | --- |
| Collection Time (hr) | Analytes | Fosmetpantotenate | | | PPA | | | PA | | | |
|  | Animal No. | Conc. (nM) | Mean Conc. (nM) | SD | Conc. (nM) | Mean Conc. (nM) | SD | Conc. (nM) | Mean Conc. (nM) | SD | |
| 0.033 | 34 | BQL | BQL | ND | 246.64 | 814.79 | 599.72 | 1012.59 | 1350.12 | 296.43 | |
|  | 35 | BQL |  |  | 755.97 |  |  | 1469.62 |  |  | |
|  | 36 | BQL |  |  | 1441.75 |  |  | 1568.14 |  |  | |
| 0.083 | 37 | BQL | BQL | ND | 2807.30 | 1912.97 | 799.05 | 2405.58 | 2114.58 | 324.16 | |
|  | 38 | BQL |  |  | 1662.32 |  |  | 2172.96 |  |  | |
|  | 39 | BQL |  |  | 1269.30 |  |  | 1765.19 |  |  | |
| 0.167 | 40 | BQL | BQL | ND | 2245.84 | 3296.57 | 2209.33 | 1866.45 | 1934.87 | 712.65 | |
|  | 41 | BQL |  |  | 5835.17 |  |  | 2679.26 |  |  | |
|  | 42 | BQL |  |  | 1808.70 |  |  | 1258.89 |  |  | |
| 0.33 | 43 | BQL | BQL | ND | 3268.50 | 4130.74 | 775.84 | 1584.56 | 1697.68 | 275.27 | |
|  | 44 | BQL |  |  | 4772.41 |  |  | 1496.99 |  |  | |
|  | 45 | BQL |  |  | 4351.31 |  |  | 2011.49 |  |  | |
| 0.5 | 46 | BQL | 3.31 | ND | 2426.31 | 2179.67 | 376.30 | 1981.39 | 1863.71 | 101.92 | |
|  | 47 | BQL |  |  | 2366.15 |  |  | 1803.50 |  |  | |
|  | 48 | 3.31 |  |  | 1746.54 |  |  | 1806.24 |  |  | |
| 1 | 49 | BQL | BQL | ND | 1026.67 | 1424.37 | 514.33 | 1937.60 | 2637.29 | 761.46 | |
|  | 50 | BQL |  |  | 1241.23 |  |  | 3448.28 |  |  | |
|  | 51 | BQL |  |  | 2005.21 |  |  | 2526.00 |  |  | |
| 2 | 52 | BQL | BQL | ND | 320.83 | 314.82 | 99.40 | 3229.34 | 2446.64 | 678.51 | |
|  | 53 | BQL |  |  | 212.55 |  |  | 2025.18 |  |  | |
|  | 54 | BQL |  |  | 411.07 |  |  | 2085.39 |  |  | |
| 4 | 55 | 2.04 | 2.04 | ND | 128.13 | 89.16 | 38.32 | 4324.03 | 3612.48 | 663.62 | |
|  | 56 | BQL |  |  | 51.53 |  |  | 3010.40 |  |  | |
|  | 57 | BQL |  |  | 87.83 |  |  | 3503.01 |  |  | |
| 8 | 58 | BQL | BQL | ND' | 37.30 | 37.36 | 9.93 | 2665.57 | 2586.21 | 686.27 | |
|  | 59 | BQL |  |  | 47.32 |  |  | 3229.34 |  |  | |
|  | 60 | BQL |  |  | 27.47 |  |  | 1863.71 |  |  | |
| (a) PA PK parameters adjusted for basal levels=(PK value in RE-024-dosed group) - (basal PA PK from control [vehicle]-dosed group). Basal C_max_=2280 nM, and basal AUC=14300 nM*hr. | | | | | | | | | | |  |
| BQL: below quantitation limit: fosmetpantotenate <1.26 nM, PPA <10.0 nM, PA <2.74 nM | | | | | | | | | | |  |
|  | | | | | | | | | | |  |

| **Blood concentrations and PK parameters of fosmetpantotenate, PPA, and PA after oral treatment with fosmetpantotenate at  300 mg/kg in male Sprague-Dawley Rats** | | | | | | | | | | |  |
| --- | --- | --- | --- | --- | --- | --- | --- | --- | --- | --- | --- |
| Collection Time (hr) | Analytes | Fosmetpantotenate | | | PPA | | | PA | | |  |
|  | Animal No. | Conc. (nM) | Mean Conc. (nM) | SD | Conc. (nM) | Mean Conc. (nM) | SD | Conc. (nM) | Mean Conc. (nM) | SD |  |
| 0.033 | 64 | 3.9 | 4.03 | ND | 6837.78 | 3663.53 | 2922.35 | 2736.73 | 1911.15 | 789.67 |  |
|  | 65 | 4.15 |  |  | 3067.98 |  |  | 1163.11 |  |  |  |
|  | 66 | BQL |  |  | 1084.82 |  |  | 1833.61 |  |  |  |
| 0.083 | 67 | BQL | BQL | ND | 2065.37 | 2702.36 | 2222.16 | 1362.89 | 1539.86 | 407.58 |  |
|  | 68 | BQL |  |  | 5173.45 |  |  | 2006.02 |  |  |  |
|  | 69 | BQL |  |  | 868.26 |  |  | 1250.68 |  |  |  |
| 0.167 | 70 | 8.83 | 18.08 | 8.01 | 23260.48 | 21930.35 | 10082.06 | 2900.93 | 3621.60 | 698.98 |  |
|  | 71 | 22.89 |  |  | 31281.33 |  |  | 4296.66 |  |  |  |
|  | 72 | 22.51 |  |  | 11249.25 |  |  | 3667.21 |  |  |  |
| 0.33 | 73 | BQL | 36.27 | ND | 20052.14 | 23862.04 | 3420.64 | 3284.07 | 4752.78 | 2288.13 |  |
|  | 74 | 64.88 |  |  | 26669.34 |  |  | 7389.16 |  |  |  |
|  | 75 | 7.67 |  |  | 24864.65 |  |  | 3585.11 |  |  |  |
| 0.5 | 76 | 2.28 | 290.11 | ND | 20854.22 | 13795.87 | 7434.15 | 3530.38 | 3448.27 | 166.47 |  |
|  | 77 | BQL |  |  | 6035.69 |  |  | 3557.74 |  |  |  |
|  | 78 | 577.94 |  |  | 14497.69 |  |  | 3256.70 |  |  |  |
| 1 | 79 | BQL | 6.98 | ND | 5694.81 | 5514.34 | 496.47 | 5117.68 | 5281.88 | 698.81 |  |
|  | 80 | BQL |  |  | 5895.33 |  |  | 6048.17 |  |  |  |
|  | 81 | 6.98 |  |  | 4952.88 |  |  | 4679.80 |  |  |  |
| 2 | 82 | 3.41 | 3.41 | ND | 2003.21 | 2599.43 | 1083.43 | 9222.77 | 6996.90 | 1940.06 |  |
|  | 83 | BQL |  |  | 3850.01 |  |  | 5665.02 |  |  |  |
|  | 84 | BQL |  |  | 1945.06 |  |  | 6102.90 |  |  |  |
| 4 | 85 | BQL | 3.69 | ND | 830.16 | 580.18 | 222.68 | 8483.85 | 7024.27 | 1337.73 |  |
|  | 86 | BQL |  |  | 403.05 |  |  | 6732.35 |  |  |  |
|  | 87 | 3.69 |  |  | 507.32 |  |  | 5856.60 |  |  |  |
| 8 | 88 | BQL | BQL | ND | 88.23 | 102.20 | 56.55 | 4433.50 | 4333.15 | 730.42 |  |
|  | 89 | BQL |  |  | 53.94 |  |  | 3557.74 |  |  |  |
|  | 90 | BQL |  |  | 164.43 |  |  | 5008.21 |  |  |  |
| (a) PA PK parameters adjusted for basal levels=(PK value in fosmetpantotenate-dosed group) - (basal PA PK from control [vehicle]-dosed group). Basal C_max_=2280 nM, and basal AUC=14300 nM*hr. | | | | | | | | | | | |
| BQL: fosmetpantotenate <1.26 nM, PPA <10.0 nM, PA <2.74 nM | | | | | | | | | | | |
|  | | | | | | | | | | | |

| **Blood concentrations and PK parameters of fosmetpantotenate, PPA, and PA after oral treatment with fosmetpantotenate at  700 mg/kg in male Sprague-Dawley Rats** | | | | | | | | | | |  |
| --- | --- | --- | --- | --- | --- | --- | --- | --- | --- | --- | --- |
| Collection Time (hr) | Analytes | Fosmetpantotenate | | | PPA | | | PA | | |  |
|  | Animal No. | Conc. (nM) | Mean Conc. (nM) | SD | Conc. (nM) | Mean Conc. (nM) | SD | Conc. (nM) | Mean Conc. (nM) | SD |  |
| 0.033 | 94 | 2149.90(a) | 114.07 | ND | 2446.36 | 1519.95 | 996.79 | 1379.31 | 1316.37 | 328.85 |  |
|  | 95 | BQL |  |  | 465.21 |  |  | 960.59 |  |  |  |
|  | 96 | 114.07 |  |  | 1648.29 |  |  | 1609.20 |  |  |  |
| 0.083 | 97 | BQL | 260.06 | ND | 1175.06 | 4876.68 | 5157.73 | 1289.00 | 1787.08 | 990.98 |  |
|  | 98 | 1.62 |  |  | 2686.99 |  |  | 1143.95 |  |  |  |
|  | 99 | 518.51 |  |  | 10768.00 |  |  | 2928.30 |  |  |  |
| 0.167 | 100 | 28.96 | 138.42 | ND | 4351.31 | 6904.62 | 2373.39 | 2142.86 | 2622.70 | 503.68 |  |
|  | 101 | 23269.54(a) |  |  | 9043.51 |  |  | 2578.00 |  |  |  |
|  | 102 | 247.87 |  |  | 7319.03 |  |  | 3147.24 |  |  |  |
| 0.33 | 103 | 2541.94 | 1291.08 | 1244.03 | 24463.61 | 19357.00 | 6867.25 | 3776.68 | 3186.46 | 1022.29 |  |
|  | 104 | 1277.3 |  |  | 22057.35 |  |  | 3776.68 |  |  |  |
|  | 105 | 54 |  |  | 11550.03 |  |  | 2006.02 |  |  |  |
| 0.5 | 106 | 509.65 | 222.37 | 248.87 | 35492.28 | 20192.50 | 14490.12 | 3503.01 | 3430.03 | 307.60 |  |
|  | 107 | 72.84 |  |  | 6677.36 |  |  | 3092.50 |  |  |  |
|  | 108 | 84.61 |  |  | 18407.86 |  |  | 3694.58 |  |  |  |
| 1 | 109 | 73.35 | 117.4 | 84.65 | 3729.70 | 3241.76 | 710.74 | 4789.27 | 4716.29 | 796.16 |  |
|  | 110 | 63.86 |  |  | 2426.31 |  |  | 3886.15 |  |  |  |
|  | 111 | 214.99 |  |  | 3569.28 |  |  | 5473.45 |  |  |  |
| 2 | 112 | 662.68 | 849.21 | ND | 1159.01 | 1369.56 | 219.01 | 5719.76 | 6467.80 | 903.26 |  |
|  | 113 | BQL |  |  | 1353.52 |  |  | 7471.26 |  |  |  |
|  | 114 | 1035.75 |  |  | 1596.15 |  |  | 6212.37 |  |  |  |
| 4 | 115 | 419.86 | 225.99 | ND | 1654.30 | 1158.35 | 450.03 | 8648.06 | 10171.50 | 1362.97 |  |
|  | 116 | BQL |  |  | 776.02 |  |  | 10591.13 |  |  |  |
|  | 117 | 32.12 |  |  | 1044.72 |  |  | 11275.31 |  |  |  |
| 8 | 118 | BQL | BQL | NA | 314.82 | 286.75 | 64.89 | 4296.66 | 4232.80 | 400.66 |  |
|  | 119 | BQL |  |  | 212.55 |  |  | 3804.05 |  |  |  |
|  | 120 | BQL |  |  | 332.87 |  |  | 4597.70 |  |  |  |
| (a) Concentration was inconsistent with cohort and not included in the mean concentration calculation. | | | | | | | | | | | |
| (b) PA PK parameters adjusted for basal levels=(PK value in fosmetpantotenate-dosed group) - (basal PA PK from control [vehicle]-dosed group). Basal C_max_=2280 nM, and basal AUC=14300 nM*hr. | | | | | | | | | | | |
| BQL: fosmetpantotenate <1.26 nM, PPA <10.0 nM, PA <2.74 nM | | | | | | | | | | | |
|  | | | | | | | | | | | |

**Pharmacokinetic measurements in Cynomolgus Monkeys**

**Blood concentrations and PK parameters of fosmetpantotenate, PPA, and PA after oral treatment with fosmetpantotenate at 300 mg/kg in male cynomolgus monkeys**

| Collection Time (hr) | Fosmetpantotenate | | | PPA | | |
| --- | --- | --- | --- | --- | --- | --- |
|  |  |  |  |  |  |  |
|  | Cyno #1 | Cyno #2 | Mean Conc. (nM) | Cyno #1 | Cyno #2 | Mean Conc. (nM) |
| 0 | BQL | BQL | BQL | BQL | BQL | BQL |
| 0.033 | BQL | BQL | BQL | BQL | BQL | BQL |
| 0.083 | 41.90 | 351.40 | 196.70 | BQL | BQL | BQL |
| 0.167 | 93.80 | 1717.40 | 905.60 | BQL | BQL | BQL |
| 0.333 | 127.50 | 972.10 | 549.80 | BQL | BQL | BQL |
| 0.5 | 211.00 | 540.80 | 375.90 | BQL | BQL | BQL |
| 1 | 9945.20 | 27417.60 | 18681.40 | 138.20 | 17.00 | 77.60 |
| 2 | 3456.30 | 22902.80 | 13179.60 | 92.60 | 107.90 | 100.30 |
| 4 | 230.80 | 267.30 | 249.10 | 114.30 | 346.90 | 230.60 |
| 8 | BQL | BQL | BQL | 274.70 | 371.00 | 322.80 |

| Collection Time (hr) | PA | | | | | |
| --- | --- | --- | --- | --- | --- | --- |
|  | Total | | | Adjusted for basal contribution | | |
|  | Cyno #1 | Cyno #2 | Mean Conc. (nM) | Cyno #1 | Cyno #2 | Mean Conc. (nM) |
| 0 | 25.60 | 49.00 | 37.30 | 0.00 | 0.00 | 0.00 |
| 0.033 | 29.30 | 43.80 | 36.50 | 3.70 | -5.20 (a) | -0.75 (a) |
| 0.083 | 24.20 | 46.50 | 35.40 | -1.40 (a) | -2.50 (a) | -1.95 (a) |
| 0.167 | 30.10 | 60.80 | 45.40 | 4.50 | 11.80 | 8.15 |
| 0.333 | 28.50 | 44.60 | 36.50 | 2.90 | -4.40 (a) | -0.75 (a) |
| 0.5 | 26.00 | 51.70 | 38.90 | 0.40 | 2.70 | 1.55 |
| 1 | 257.00 | 130.50 | 193.80 | 231.40 | 81.50 | 156.45 |
| 2 | 509.00 | 350.30 | 429.70 | 483.40 | 301.30 | 392.35 |
| 4 | 489.90 | 667.80 | 578.80 | 464.30 | 618.80 | 541.55 |
| 8 | 342.10 | 744.40 | 543.20 | 316.50 | 695.40 | 505.95 |

1. Treated as 0 for calculations.

BQL: below quantitation limit; NC: not calculated due to insufficient data points; ND: not determined; PA: pantothenate; PPA: phosphopantothenate
