# Supplementary material for: Fosmetpantotenate (RE-024), a phosphopantothenate replacement therapy for pantothenate kinase-associated neurodegeneration: Mechanism of action and efficacy in nonclinical models
Source: PLoS One. 2018 Mar 9;13(3):e0192028. doi: 10.1371/journal.pone.0192028 (PMC5844530; doi:10.1371/journal.pone.0192028)
Supplement: S5 Table — (DOCX) [file pone.0192028.s007.docx]

**S5 Table. Metabolite concentrations after dosing fosmetpantotenate PO in CD-1 mice, Sprague-Dawley rats, and cynomolgus monkeys**

**Compound 1**

Concentrations of compound 1 in whole blood of CD-1 mice after a single dose of fosmetpantotenate at the indicated level.

| Mice | Compound 1: 100 mg/kg  RE-024-02 by PO | | |  | Mice | Compound 1: 300 mg/kg  RE-024-02 by PO | | |  | Mice | Compound 1: 700 mg/kg  RE-024-02 by PO | | |
| --- | --- | --- | --- | --- | --- | --- | --- | --- | --- | --- | --- | --- | --- |
| Time | Animal No. | Whole Blood ^(a)^ | |  | Time | Animal No. | Whole Blood ^(a)^ | |  | Time | Animal No. | Whole Blood^(a)^ | |
| (hr) |  | Conc. (ng/mL) | Conc.  (nM) |  | (hr) |  | Conc. (ng/mL) | Conc.  (nM) |  | (hr) |  | Conc. (ng/mL) | Conc.  (nM) |
|  |  |  |  |  |  |  |  |  |  |  |  |  |  |
| 0.033 | 35 | 4.6 | 12.2 |  | 0.033 | 75 | 8.7 | 22.9 |  | 0.033 | 115 | 22.6 | 59.3 |
|  | 36 | 4 | 10.6 |  |  | 76 | 70.8 | 186.2 |  |  | 116 | 66.6 | 175.1 |
|  | 37 | 74.4 | 195.6 |  |  | 77 | 52.7 | 138.7 |  |  | 117 | 198 | 520.6 |
|  | 38 | 3.2 | 8.4 |  |  | 78 | 7.2 | 18.9 |  |  | 118 | 119.4 | 313.9 |
| 0.083 | 39 | BQL | BQL |  | 0.083 | 79 | 1.6 | 4.2 |  | 0.083 | 119 | 165 | 433.8 |
|  | 40 | 1.4 | 3.8 |  |  | 80 | BQL | BQL |  |  | 120 | 2.2 | 5.9 |
|  | 41 | 1 | 2.7 |  |  | 81 | 16.9 | 44.3 |  |  | 121 | 624 | 1640.7 |
|  | 42 | BQL | BQL |  |  | 82 | BQL | BQL |  |  | 122 | 75 | 197.2 |
| 0.167 | 43 | 4.3 | 11.4 |  | 0.167 | 83 | 21.9 | 57.6 |  | 0.167 | 123 | 636 | 1672.2 |
|  | 44 | BQL | BQL |  |  | 84 | 24.1 | 63.3 |  |  | 124 | 235.8 | 620 |
|  | 45 | 10.8 | 28.4 |  |  | 85 | 53.3 | 140.1 |  |  | 125 | 165 | 433.8 |
|  | 46 | BQL | BQL |  |  | 86 | 17.7 | 46.5 |  |  | 126 | 720 | 1893.1 |
| 0.333 | 47 | BQL | BQL |  | 0.333 | 87 | 2.4 | 6.2 |  | 0.333 | 127 | 108.6 | 285.5 |
|  | 48 | BQL | BQL |  |  | 88 | 4.3 | 11.3 |  |  | 128 | 63.6 | 167.2 |
|  | 49 | BQL | BQL |  |  | 89 | 3.8 | 10 |  |  | 129 | NS | NS |
|  | 50 | 0.8 | 2 |  |  | 90 | 1.5 | 3.9 |  |  | 130 | NS | NS |
| 0.5 | 51 | BQL | BQL |  | 0.5 | 91 | 5 | 13.1 |  | 0.5 | 131 | 12 | 31.6 |
|  | 52 | BQL | BQL |  |  | 92 | 4.4 | 11.5 |  |  | 132 | 41.6 | 109.3 |
|  | 53 | BQL | BQL |  |  | 93 | 4.5 | 11.9 |  |  | 133 | 9.4 | 24.8 |
|  | 54 | BQL | BQL |  |  | 94 | 2.8 | 7.4 |  |  | 134 | 22.9 | 60.1 |
| 1 | 55 | BQL | BQL |  | 1 | 95 | BQL | BQL |  | 1 | 135 | 48.8 | 128.4 |
|  | 56 | BQL | BQL |  |  | 96 | 0.8 | 2.1 |  |  | 136 | 16 | 42.1 |
|  | 57 | BQL | BQL |  |  | 97 | BQL | BQL |  |  | 137 | 6.1 | 15.9 |
|  | 58 | BQL | BQL |  |  | 98 | BQL | BQL |  |  | 138 | 8.8 | 23.2 |
| 2 | 59 | BQL | BQL |  | 2 | 99 | BQL | BQL |  | 2 | 139 | 5.5 | 14.6 |
|  | 60 | BQL | BQL |  |  | 100 | BQL | BQL |  |  | 140 | 4 | 10.4 |
|  | 61 | BQL | BQL |  |  | 101 | BQL | BQL |  |  | 141 | 5.6 | 14.8 |
|  | 62 | BQL | BQL |  |  | 102 | BQL | BQL |  |  | 142 | 10.2 | 26.8 |
| 4 | 63 | BQL | BQL |  | 4 | 103 | BQL | BQL |  | 4 | 143 | BQL | BQL |
|  | 64 | BQL | BQL |  |  | 104 | BQL | BQL |  |  | 144 | 0.8 | 2.1 |
|  | 65 | BQL | BQL |  |  | 105 | BQL | BQL |  |  | 145 | BQL | BQL |
|  | 66 | BQL | BQL |  |  | 106 | BQL | BQL |  |  | 146 | BQL | BQL |
| 8 | 67 | BQL | BQL |  | 8 | 107 | BQL | BQL |  | 8 | 147 | BQL | BQL |
|  | 68 | BQL | BQL |  |  | 108 | BQL | BQL |  |  | 148 | BQL | BQL |
|  | 69 | BQL | BQL |  |  | 109 | BQL | BQL |  |  | 149 | BQL | BQL |
|  | 70 | BQL | BQL |  |  | 110 | BQL | BQL |  |  | 150 | BQL | BQL |

1. The LLOQ for Compound 1 in whole blood was 0.6 ng/mL (1.6 nM).

Concentrations of compound 1 in whole blood of Sprague-Dawley rats after a single dose of fosmetpantotenate at the indicated level.

| Rats | Compound 1: 100 mg/kg  RE-024-02 by PO | | |  | Rats | Compound 1: 300 mg/kg  RE-024-02 by PO | | |  | Rats | Compound 1: 700 mg/kg  RE-024-02 by PO | | |
| --- | --- | --- | --- | --- | --- | --- | --- | --- | --- | --- | --- | --- | --- |
|  |  | Whole Blood | |  |  |  | Whole Blood |  |  |  |  | Whole Blood |  |
| Time | Animal | Conc. (ng/mL) | Conc. (nM) |  | Time | Animal | Conc. (ng/mL) | Conc. (nM) |  | Time | Animal | Conc. (ng/mL) | Conc. (nM) |
| 0.033 | 34 | BQL | BQL |  | 0.033 | 64 | 40.98 | 107.78 |  | 0.033 | 94 | 362.4 | 952.86 |
|  | 35 | 0.73 | 1.91 |  |  | 65 | 14.64 | 38.49 |  |  | 95 | 16.26 | 42.75 |
|  | 36 | 3.1 | 8.14 |  |  | 66 | 8.7 | 22.87 |  |  | 96 | 45.48 | 119.58 |
| 0.083 | 37 | 11.46 | 30.13 |  | 0.083 | 67 | 6.3 | 16.56 |  | 0.083 | 97 | 19.98 | 52.53 |
|  | 38 | 2.83 | 7.45 |  |  | 68 | 15.84 | 41.65 |  |  | 98 | 12.72 | 33.44 |
|  | 39 | 1.09 | 2.87 |  |  | 69 | BQL | BQL |  |  | 99 | 226.2 | 594.75 |
| 0.167 | 40 | BQL | BQL |  | 0.167 | 70 | 11001 | 57.9 |  | 0.167 | 100 | 76.2 | 200.35 |
|  | 41 | 3.88 | 10.19 |  |  | 71 | 72 | 189.31 |  |  | 101 | 972 | 2555.68 |
|  | 42 | 6.3 | 16.56 |  |  | 72 | 35.22 | 92.6 |  |  | 102 | 130.8 | 343.91 |
| 0.33 | 43 | BQL | BQL |  | 0.33 | 73 | 22.56 | 59.32 |  | 0.33 | 103 | 242.4 | 637.34 |
|  | 44 | 1.21 | 3.18 |  |  | 74 | 41.34 | 108.7 |  |  | 104 | 111.6 | 293.43 |
|  | 45 | 1.42 | 3.72 |  |  | 75 | 21.6 | 56.79 |  |  | 105 | 42.54 | 111.85 |
| 0.5 | 46 | BQL | BQL |  | 0.5 | 76 | 7.14 | 18.77 |  | 0.5 | 106 | 79.2 | 108.24 |
|  | 47 | BQL | BQL |  |  | 77 | 6.18 | 16.25 |  |  | 107 | 28.2 | 74.15 |
|  | 48 | BQL | BQL |  |  | 78 | 23.64 | 62.16 |  |  | 108 | 12.84 | 33.76 |
| 1 | 49 | BQL | BQL |  | 1 | 79 | BQL | BQL |  | 1 | 109 | 26.1 | 68.62 |
|  | 50 | BQL | BQL |  |  | 80 | 3.38 | 8.73 |  |  | 110 | 11.82 | 31.08 |
|  | 51 | BQL | BQL |  |  | 81 | 12.12 | 31.87 |  |  | 111 | 27.06 | 71.15 |
| 2 | 52 | BQL | BQL |  | 2 | 82 | BQL | BQL |  | 2 | 112 | 26.64 | 10.04 |
|  | 53 | BQL | BQL |  |  | 83 | BQL | BQL |  |  | 113 | 0.87 | 2.29 |
|  | 54 | BQL | BQL |  |  | 84 | BQL | BQL |  |  | 114 | 40.62 | 106.8 |
| 4 | 55 | BQL | BQL |  | 4 | 85 | BQL | BQL |  | 4 | 115 | 21.66 | 56.96 |
|  | 56 | BQL | BQL |  |  | 86 | BQL | BQL |  |  | 116 | BQL | BQL |
|  | 57 | BQL | BQL |  |  | 87 | BQL | BQL |  |  | 117 | 5.88 | 15.46 |
| 8 | 58 | BQL | BQL |  | 8 | 88 | BQL | BQL |  | 8 | 118 | BQL | BQL |
|  | 59 | BQL | BQL |  |  | 89 | BQL | BQL |  |  | 119 | BQL | BQL |
|  | 60 | BQL | BQL |  |  | 90 | BQL | BQL |  |  | 120 | BQL | BQL |

1. The LLOQ for Compound 1 in whole blood was 0.60 ng/mL (1.58 nM).

Concentrations of compound 1 in whole blood of cynomolgus monkeys after a single oral dose (300 mg/kg) of fosmetpantotenate

| Time Point (hr) | Concentration (nM) ^(a)^ | |
| --- | --- | --- |
|  | Animal 7 | Animal 8 |
| 0.083 | 17.2 | 11.28 |
| 0.25 | 175.11 | 34.71 |
| 0.5 | 2479.64 | 3160.36 |
| 1 | 1801.91 | 6028.71 |
| 2 | 4069.2 | 612.42 |
| 4 | 54.58 | 96.23 |
| 8 | 7.89 | 4.57 |
| 24 | 24 | 9.15 |

1. Data represent the sum of the individual compound 1 diastereomers. The LLOQ for the sum of the diastereomers in whole blood was
   0.60 ng/mL (1.58 nM).

**Compound 2**

Concentrations of compound 2 in whole blood of CD-1 mice after a single dose of fosmetpantotenate at the indicated level.

| Mice | Compound 2: 100 mg/kg  RE-024-02 by PO | | |  | Mice | Compound 2: 300 mg/kg  RE-024-02 by PO | | |  | Mice | Compound 2: 700 mg/kg  RE-024-02 by PO | | | |
| --- | --- | --- | --- | --- | --- | --- | --- | --- | --- | --- | --- | --- | --- | --- |
| Time | Animal No. | Whole Blood^(a)^ | |  | Time | Animal No. | Whole Blood^(a)^ | |  | Time | Animal No. | Whole Blood^(a)^ | | |
| (hr) |  | Conc. (ng/mL) | Conc.  (nM) |  | (hr) |  | Conc. (ng/mL) | Conc.  (nM) |  | (hr) |  | Conc. (ng/mL) | Conc.  (nM) |  |
|  |  |  |  |  |  |  |  |  |  |  |  |  |  |  |
| 0.033 | 35 | 295.8 | 1001.9 |  | 0.033 | 75 | 43.9 | 148.8 |  | 0.033 | 115 | 1146 | 3881.7 |  |
|  | 36 | 111 | 376 |  |  | 76 | 3780 | 12803.6 |  |  | 116 | 3456 | 11706.1 |  |
|  | 37 | 2856 | 9673.8 |  |  | 77 | 4326 | 14653 |  |  | 117 | 3648 | 12356.5 |  |
|  | 38 | 160.2 | 542.6 |  |  | 78 | 307.2 | 1040.5 |  |  | 118 | 3852 | 13047.5 |  |
| 0.083 | 39 | BQL | BQL |  | 0.083 | 79 | 57.1 | 193.3 |  | 0.083 | 119 | 2382 | 8068.3 |  |
|  | 40 | 17.4 | 58.9 |  |  | 80 | 9.2 | 31.3 |  |  | 120 | 95.4 | 323.1 |  |
|  | 41 | 27.6 | 93.5 |  |  | 81 | 154.2 | 522.3 |  |  | 121 | 9960 | 33736.4 |  |
|  | 42 | BQL | BQL |  |  | 82 | BQL | BQL |  |  | 122 | 1980 | 6706.6 |  |
| 0.167 | 43 | 32.9 | 111.6 |  | 0.167 | 83 | 499.2 | 1690.9 |  | 0.167 | 123 | 4374 | 14815.6 |  |
|  | 44 | 41 | 138.8 |  |  | 84 | 738 | 2499.7 |  |  | 124 | 2448 | 8291.8 |  |
|  | 45 | 250.2 | 847.5 |  |  | 85 | 1722 | 5832.7 |  |  | 125 | 2892 | 9795.8 |  |
|  | 46 | 19.7 | 66.7 |  |  | 86 | 464.4 | 1573 |  |  | 126 | 7080 | 23981.3 |  |
| 0.333 | 47 | 32.1 | 108.7 |  | 0.333 | 87 | 442.8 | 1499.8 |  | 0.333 | 127 | 1908 | 6462.8 |  |
|  | 48 | 36.2 | 122.8 |  |  | 88 | 495 | 1676.7 |  |  | 128 | 1656 | 5609.2 |  |
|  | 49 | 25.5 | 86.4 |  |  | 89 | 550.2 | 1863.6 |  |  | 129 | NS | NS |  |
|  | 50 | 84 | 284.5 |  |  | 90 | 391.2 | 1325.1 |  |  | 130 | NS | NS |  |
| 0.5 | 51 | 14 | 47.4 |  | 0.5 | 91 | 253.8 | 859.7 |  | 0.5 | 131 | 463.8 | 1571 |  |
|  | 52 | 36.8 | 124.8 |  |  | 92 | 472.2 | 1599.4 |  |  | 132 | 1002 | 3394 |  |
|  | 53 | 22.3 | 75.6 |  |  | 93 | 328.2 | 1111.7 |  |  | 133 | 648 | 2194.9 |  |
|  | 54 | 39.2 | 132.9 |  |  | 94 | 282 | 955.2 |  |  | 134 | 1272 | 4308.5 |  |
| 1 | 55 | 12.8 | 43.5 |  | 1 | 95 | 108.6 | 367.8 |  | 1 | 135 | 541.2 | 1833.1 |  |
|  | 56 | 3.2 | 10.7 |  |  | 96 | 130.8 | 443 |  |  | 136 | 318 | 1077.1 |  |
|  | 57 | 8.9 | 30.1 |  |  | 97 | 97.2 | 329.2 |  |  | 137 | 373.2 | 1264.1 |  |
|  | 58 | 28.5 | 96.5 |  |  | 98 | 42.1 | 142.5 |  |  | 138 | 381 | 1290.5 |  |
| 2 | 59 | BQL | BQL |  | 2 | 99 | 19.4 | 65.6 |  | 2 | 139 | 432.6 | 1465.3 |  |
|  | 60 | BQL | BQL |  |  | 100 | 44.5 | 150.6 |  |  | 140 | 187.2 | 634.1 |  |
|  | 61 | 5.1 | 17.3 |  |  | 101 | 23 | 78 |  |  | 141 | 331.2 | 1121.8 |  |
|  | 62 | 8.8 | 29.7 |  |  | 102 | 25 | 84.7 |  |  | 142 | 331.8 | 1123.9 |  |
| 4 | 63 | BQL | BQL |  | 4 | 103 | 21.9 | 74.2 |  | 4 | 143 | 104.4 | 353.6 |  |
|  | 64 | 3.5 | 11.7 |  |  | 104 | 15.4 | 52.2 |  |  | 144 | 84 | 284.5 |  |
|  | 65 | BQL | BQL |  |  | 105 | 22.8 | 77.2 |  |  | 145 | 92.4 | 313 |  |
|  | 66 | BQL | BQL |  |  | 106 | 26.2 | 88.6 |  |  | 146 | 88.2 | 298.8 |  |
| 8 | 67 | BQL | BQL |  | 8 | 107 | 16.3 | 55.3 |  | 8 | 147 | 68.4 | 231.7 |  |
|  | 68 | 3.5 | 11.9 |  |  | 108 | 9.4 | 31.7 |  |  | 148 | 27.8 | 94.1 |  |
|  | 69 | 3.7 | 12.6 |  |  | 109 | 8 | 27 |  |  | 149 | 42.4 | 143.5 |  |
|  | 70 | BQL | BQL |  |  | 110 | 13 | 43.9 |  |  | 150 | 44 | 149 |  |

1. The LLOQ for Compound 2 in whole blood was 3 ng/mL (10.2 nM).

Concentrations of compound 2 in whole blood of Sprague-Dawley rats after a single dose of fosmetpantotenate at the indicated level.

| Rats | Compound 2: 100 mg/kg  RE-024-02 by PO | | |  | Rats | Compound 2: 300 mg/kg  RE-024-02 by PO | | |  | Rats | Compound 2: 700 mg/kg  RE-024-02 by PO | | |
| --- | --- | --- | --- | --- | --- | --- | --- | --- | --- | --- | --- | --- | --- |
|  |  | Whole Blood | |  |  |  | Whole Blood | |  |  |  | Whole Blood | |
| Time | Animal | Conc. (ng/mL) | Conc. (nM) |  | Time | Animal | Conc. (ng/mL) | Conc. (nM) |  | Time | Animal | Conc. (ng/mL) | Conc. (nM) |
| 0.033 | 34 | BQL | BQL |  | 0.033 | 64 | 325.80 | 1103.55 |  | 0.033 | 94 | 1056.00 | 3576.87 |
|  | 35 | 10.02 | 33.94 |  |  | 65 | 142.80 | 483.69 |  |  | 95 | 60.00 | 203.23 |
|  | 36 | 23.22 | 78.65 |  |  | 66 | 36.72 | 124.38 |  |  | 96 | 332.40 | 1125.90 |
| 0.083 | 37 | 123.00 | 416.62 |  | 0.083 | 67 | 65.40 | 221.52 |  | 0.083 | 97 | 73.20 | 247.94 |
|  | 38 | 37.08 | 125.60 |  |  | 68 | 101.40 | 343.46 |  |  | 98 | 75.00 | 254.04 |
|  | 39 | 16.38 | 55.48 |  |  | 69 | 6.66 | 22.56 |  |  | 99 | 798.00 | 2702.98 |
| 0.167 | 40 | 4.48 | 15.17 |  | 0.167 | 70 | 133.20 | 451.17 |  | 0.167 | 100 | 286.20 | 969.41 |
|  | 41 | 36.06 | 122.14 |  |  | 71 | 261.60 | 886.09 |  |  | 101 | 2940.00 | 9958.34 |
|  | 42 | 36.06 | 122.14 |  |  | 72 | 543.00 | 1839.24 |  |  | 102 | 948.00 | 3211.06 |
| 0.33 | 43 | 11.76 | 39.83 |  | 0.33 | 73 | 249.60 | 845.44 |  | 0.33 | 103 | 1380.00 | 4674.32 |
|  | 44 | 37.14 | 125.80 |  |  | 74 | 708.00 | 2398.13 |  |  | 104 | 804.00 | 2723.30 |
|  | 45 | 20.58 | 69.71 |  |  | 75 | 174.60 | 591.40 |  |  | 105 | 220.20 | 745.86 |
| 0.5 | 46 | 23.22 | 78.65 |  | 0.5 | 76 | 183.00 | 619.86 |  | 0.5 | 106 | 452.40 | 1532.36 |
|  | 47 | 12.78 | 43.29 |  |  | 77 | 70.80 | 239.81 |  |  | 107 | 247.20 | 837.31 |
|  | 48 | 11.16 | 37.80 |  |  | 78 | 486.00 | 1646.17 |  |  | 108 | 285.00 | 965.35 |
| 1 | 49 | 15.42 | 52.23 |  | 1 | 79 | 51.84 | 175.59 |  | 1 | 109 | 244.80 | 829.18 |
|  | 50 | 17.16 | 58.12 |  |  | 80 | 98.40 | 333.30 |  |  | 110 | 121.20 | 410.53 |
|  | 51 | 7.74 | 26.22 |  |  | 81 | 177.00 | 599.53 |  |  | 111 | 314.40 | 1064.93 |
| 2 | 52 | 6.60 | 22.36 |  | 2 | 82 | 76.20 | 258.10 |  | 2 | 112 | 105.00 | 355.65 |
|  | 53 | 3.01 | 10.20 |  |  | 83 | 38.46 | 130.27 |  |  | 113 | 61.80 | 209.33 |
|  | 54 | BQL | BQL |  |  | 84 | 47.10 | 159.54 |  |  | 114 | 118.20 | 400.37 |
| 4 | 55 | 5.57 | 18.87 |  | 4 | 85 | 44.22 | 149.78 |  | 4 | 115 | 188.40 | 638.15 |
|  | 56 | BQL | BQL |  |  | 86 | 17.28 | 58.53 |  |  | 116 | 70.20 | 237.78 |
|  | 57 | BQL | BQL |  |  | 87 | 10.98 | 37.19 |  |  | 117 | 158.40 | 536.53 |
| 8 | 58 | BQL | BQL |  | 8 | 88 | 9.36 | 31.70 |  | 8 | 118 | 40.38 | 136.77 |
|  | 59 | 6.42 | 21.75 |  |  | 89 | 5.25 | 17.78 |  |  | 119 | 39.66 | 134.34 |
|  | 60 | BQL | BQL |  |  | 90 | 7.14 | 24.18 |  |  | 120 | 8.88 | 30.08 |

1. The LLOQ for Compound 2 in whole blood was 3 ng/mL (10.2 nM).

Concentrations of compound 2 in whole blood of cynomolgus monkeys after a single oral dose (300 mg/kg) of fosmetpantotenate

| Time Point (hr) | Concentration (nM) ^(a)^ | |
| --- | --- | --- |
|  | Animal 7 | Animal 8 |
| 0.083 | BQL | BQL |
| 0.25 | 38.41 | 17.48 |
| 0.5 | 1855.1 | 1210.45 |
| 1 | 1974.6 | 2023.57 |
| 2 | 4859.26 | 9989.84 |
| 4 | 2346.51 | 4441.22 |
| 8 | 1145.41 | 996.85 |
| 24 | 97.35 | 700.13 |

1. The LLOQ for compound 2 in whole blood was 0.60 ng/mL (2.03 nM).

**Compound 3**

Concentrations of compound 3 in whole blood of CD-1 mice after a single dose of fosmetpantotenate at the indicated level.

| Mice | Compound 3: 100 mg/kg  RE-024-02 by PO | | |  | Mice | Compound 3: 300 mg/kg  RE-024-02 by PO | | |  | Mice | Compound 3: 700 mg/kg  RE-024-02 by PO | | |
| --- | --- | --- | --- | --- | --- | --- | --- | --- | --- | --- | --- | --- | --- |
| Time | Animal No. | Whole Blood^(a)^ | |  | Time | Animal No. | Whole Blood^(a)^ | |  | Time | Animal No. | Whole Blood^(a)^ | |
| (hr) |  | Conc. (ng/mL) | Conc.  (nM) |  | (hr) |  | Conc. (ng/mL) | Conc.  (nM) |  | (hr) |  | Conc. (ng/mL) | Conc.  (nM) |
|  |  |  |  |  |  |  |  |  |  |  |  |  |  |
| 0.033 | 35 | 115.2 | 409.7 |  | 0.033 | 75 | 54.2 | 192.7 |  | 0.033 | 115 | 243 | 864.2 |
|  | 36 | 84.6 | 300.9 |  |  | 76 | 1572 | 5590.3 |  |  | 116 | 654 | 2325.7 |
|  | 37 | 583.2 | 2074 |  |  | 77 | 1260 | 4480.8 |  |  | 117 | 425.4 | 1512.8 |
|  | 38 | 86.4 | 307.3 |  |  | 78 | 133.2 | 473.7 |  |  | 118 | 948 | 3371.3 |
| 0.083 | 39 | BQL | BQL |  | 0.083 | 79 | 74.4 | 264.6 |  | 0.083 | 119 | 798 | 2837.8 |
|  | 40 | 33 | 117.4 |  |  | 80 | 19.4 | 69.1 |  |  | 120 | 112.8 | 401.1 |
|  | 41 | 45.2 | 160.7 |  |  | 81 | 100.8 | 358.5 |  |  | 121 | 828 | 2944.5 |
|  | 42 | 10.4 | 36.9 |  |  | 82 | BQL | BQL |  |  | 122 | 428.4 | 1523.5 |
| 0.167 | 43 | 53.9 | 191.8 |  | 0.167 | 83 | 506.4 | 1800.9 |  | 0.167 | 123 | 1746 | 6209.1 |
|  | 44 | 40.1 | 142.5 |  |  | 84 | 732 | 2603.1 |  |  | 124 | 1260 | 4480.8 |
|  | 45 | 199.8 | 710.5 |  |  | 85 | 936 | 3328.6 |  |  | 125 | 1506 | 5355.6 |
|  | 46 | 61.2 | 217.6 |  |  | 86 | 267.6 | 951.6 |  |  | 126 | 2622 | 9324.3 |
| 0.333 | 47 | 34.7 | 123.3 |  | 0.333 | 87 | 409.8 | 1457.3 |  | 0.333 | 127 | 288.6 | 1026.3 |
|  | 48 | 23.2 | 82.6 |  |  | 88 | 394.8 | 1404 |  |  | 128 | 720 | 2560.5 |
|  | 49 | 40.7 | 144.9 |  |  | 89 | 292.2 | 1039.1 |  |  | 129 | NS | NS |
|  | 50 | 56.2 | 199.7 |  |  | 90 | 170.4 | 606 |  |  | 130 | NS | NS |
| 0.5 | 51 | 8.5 | 30.1 |  | 0.5 | 91 | 163.2 | 580.4 |  | 0.5 | 131 | 162 | 576.1 |
|  | 52 | 43.1 | 153.2 |  |  | 92 | 216.6 | 770.3 |  |  | 132 | 126 | 448.1 |
|  | 53 | 30.2 | 107.5 |  |  | 93 | 289.2 | 1028.4 |  |  | 133 | 156 | 554.8 |
|  | 54 | 45.7 | 162.4 |  |  | 94 | 339 | 1205.5 |  |  | 134 | 189 | 672.1 |
| 1 | 55 | 10.5 | 37.3 |  | 1 | 95 | 59.8 | 212.5 |  | 1 | 135 | 128.4 | 456.6 |
|  | 56 | 6.2 | 22.2 |  |  | 96 | 56 | 199.3 |  |  | 136 | 78 | 277.4 |
|  | 57 | 14.8 | 52.5 |  |  | 97 | 43.4 | 154.3 |  |  | 137 | 89.4 | 317.9 |
|  | 58 | 44.8 | 159.2 |  |  | 98 | 16.7 | 59.3 |  |  | 138 | 44.7 | 159 |
| 2 | 59 | 6.7 | 23.7 |  | 2 | 99 | 8.5 | 30.1 |  | 2 | 139 | 48.2 | 171.6 |
|  | 60 | BQL | BQL |  |  | 100 | 18.1 | 64.2 |  |  | 140 | 67.2 | 239 |
|  | 61 | BQL | BQL |  |  | 101 | 10.1 | 35.8 |  |  | 141 | 69.6 | 247.5 |
|  | 62 | 10.4 | 37.1 |  |  | 102 | 17.4 | 61.9 |  |  | 142 | 72 | 256 |
| 4 | 63 | BQL | BQL |  | 4 | 103 | 17.2 | 61.2 |  | 4 | 143 | 30.2 | 107.3 |
|  | 64 | BQL | BQL |  |  | 104 | 12.2 | 43.3 |  |  | 144 | 18.5 | 65.7 |
|  | 65 | BQL | BQL |  |  | 105 | 16.1 | 57.4 |  |  | 145 | 20.9 | 74.5 |
|  | 66 | BQL | BQL |  |  | 106 | 7.3 | 25.8 |  |  | 146 | 37.7 | 134.2 |
| 8 | 67 | 6.1 | 21.6 |  | 8 | 107 | 7.6 | 26.9 |  | 8 | 147 | 26.5 | 94.1 |
|  | 68 | BQL | BQL |  |  | 108 | 6.1 | 21.6 |  |  | 148 | 10.1 | 36.1 |
|  | 69 | BQL | BQL |  |  | 109 | BQL | BQL |  |  | 149 | 6.1 | 21.8 |
|  | 70 | BQL | BQL |  |  | 110 | BQL | BQL |  |  | 150 | 11.9 | 42.5 |

1. The LLOQ for compound 3 in whole blood was 6 ng/mL (21.3 nM).

Concentrations of compound 3 in whole blood of cynomolgus monkeys after a single oral dose (300 mg/kg) of fosmetpantotenate

| Time Point (hr) | Concentration (nM) ^(a)^ | |
| --- | --- | --- |
|  | Animal 7 | Animal 8 |
| 0.083 | 2.56 | 0.43 |
| 0.25 | 5.33 | 3.41 |
| 0.5 | 138.9 | 62.94 |
| 1 | 128.02 | 425.68 |
| 2 | 382.79 | 1287.91 |
| 4 | 432.29 | 654.2 |
| 8 | 618.78 | 508.25 |
| 24 | 235.56 | 1393.31 |

1. The LLOQ for compound 3 in whole blood was 0.60 ng/mL (2.13 nM).

Concentrations of compound 4 in whole blood of cynomolgus monkeys after a single oral dose (300 mg/kg) of fosmetpantotenate

| Time Point (hr) | Concentration (nM) ^(a)^ | |
| --- | --- | --- |
|  | Animal 7 | Animal 8 |
| 0.083 | 13.16 | 8.08 |
| 0.25 | 84.84 | 26.06 |
| 0.5 | 3504.71 | 1748.71 |
| 1 | 1459.02 | 11431.17 |
| 2 | 5067.85 | 5694.67 |
| 4 | 664.87 | 600.1 |
| 8 | 22.81 | 8.21 |
| 24 | 3 | 10.82 |

1. Data represent the sum of the individual compound 4 diastereomers. The LLOQ for the sum of the diastereomers in whole blood was
   0.60 ng/mL (1.30 nM).

BQL: below quantitation limit; LLOQ: lower limit of quantitation; RE-024: fosmetpantotenate
