# Supplementary material for: Fosmetpantotenate (RE-024), a phosphopantothenate replacement therapy for pantothenate kinase-associated neurodegeneration: Mechanism of action and efficacy in nonclinical models
Source: PLoS One. 2018 Mar 9;13(3):e0192028. doi: 10.1371/journal.pone.0192028 (PMC5844530; doi:10.1371/journal.pone.0192028)
Supplement: S6 Table — (DOCX) [file pone.0192028.s008.docx]

**S6 Table. Concentrations of fosmetpantotenate, PPA, and PA in dialysates and blood after a single oral dose of fosmetpantotenate in non-fasted male C57/Bl6 mice (700 mg/kg or 125 µg) or in cynomolgus monkeys (100 or 300 mg/kg).**

**Concentrations of fosmetpantotenate total in striatal dialysate after a single oral dose of fosmetpantotenate at 700 mg/kg in non-fasted male C57/Bl6 mice**

|  | Fosmetpantotenate Total Concentration (nM) in Mouse Striatal Dialysate (Corrected for Dilution) ^(a)^ | | | | | | | |  |  |
| --- | --- | --- | --- | --- | --- | --- | --- | --- | --- | --- |
| Collection Time  (hr) ^(b)^ | MD02-M14-0405 | MD02-M14-0406 | MD02-M14-0407 | MD02-M14-0408 | MD02-M14-0409 | MD02-M14-0410 | MD02-M14-0411 | MD02-M14-0412 | Mean | SD |
| 0 | BQL | BQL | BQL | BQL | BQL | BQL | BQL | BQL | BQL | ND |
| 0.5 | BQL | BQL | BQL | BQL | BQL | BQL | BQL | BQL | BQL | ND |
| 1 | BQL | BQL | BQL | BQL | BQL | BQL | BQL | BQL | BQL | ND |
| 1.5 | BQL | BQL | BQL | BQL | BQL | BQL | BQL | BQL | BQL | ND |
| 2 | BQL | BQL | BQL | BQL | BQL | BQL | BQL | BQL | BQL | ND |
| 2.5 | BQL | BQL | BQL | BQL | BQL | BQL | BQL | BQL | BQL | ND |
| 3 | BQL | BQL | BQL | BQL | BQL | BQL | BQL | BQL | BQL | ND |
| 3.5 | BQL | BQL | BQL | BQL | BQL | BQL | BQL | BQL | BQL | ND |
| 4 | BQL | BQL | BQL | BQL | BQL | BQL | BQL | BQL | BQL | ND |

1. Striatal dialysate concentrations were determined by converting measured ng/mL dialysate concentration to nM *(nM=[(ng/mL)/MW] * 1000, where MW=474.44 g/mol).* The measured values (nM) were multiplied by a factor of 7.67 to correct for dilution of the dialysate (1 part dialysate to 6.67 parts carrier plus quench).
2. Time relative to fosmetpantotenate dose: Time is the end time of the 30-min dialysate collection period.

Concentrations were BQL in all samples for both D1 (BQL=4.72 nM) and D2 (BQL=3.36 nM), resulting in a BQL of 8.08 nM for fosmetpantotenate

**Concentrations of PPA in striatal dialysate after a single oral dose of fosmetpantotenate at 700 mg/kg in non-fasted male C57/Bl6 mice**

PPA Concentration (nM) in Mouse Striatal Dialysate (Corrected for Dilution) ^(a)^

| Collection Time  (hr) ^(b)^ | MD02-M14-0405 | MD02-M14-0406 | MD02-M14-0407 | MD02-M14-0408 | MD02-M14-0409 | MD02-M14-0410 | MD02-M14-0411 | MD02-M14-0412 | Mean ^(c)^ | SD |
| --- | --- | --- | --- | --- | --- | --- | --- | --- | --- | --- |
| 0 | BQL | BQL | BQL | BQL | BQL | BQL | BQL | BQL | BQL | ND |
| 0.5 | BQL | 45.6 | 70.2 | 32.6 | 6.8 | BQL | 78.7 | BQL | 46.8 | 29 |
| 1 | BQL | 162 | 234 | 154.1 | 100.5 | 18 | 276.8 | 5.7 | 135.9 | 102.2 |
| 1.5 | BQL | 65.4 | 292.2 | 132.3 | 141.2 | 22.2 | 282 | BQL | 155.9 | 110.7 |
| 2 | BQL | 87.2 | 271.7 | 82.5 | 132.5 | 33.3 | 274.3 | BQL | 146.9 | 102.6 |
| 2.5 | BQL | 61.8 | 225.8 | 60.5 | 98.7 | 16.7 | 218.1 | BQL | 113.6 | 87.9 |
| 3 | BQL | 37.2 | 179.2 | 75.1 | 95.6 | 16 | 186.1 | BQL | 98.2 | 71.1 |
| 3.5 | BQL | 34.6 | 178.9 | 62.3 | 70.2 | BQL | 228.4 | BQL | 114.9 | 83.9 |
| 4 | BQL | 31.8 | 146.9 | 50.8 | 64.6 | 8.8 | 206.9 | BQL | 84.9 | 76 |

1. Striatal dialysate concentrations were determined by converting measured ng/mL dialysate concentration to nM *(nM=[(ng/mL) / MW] * 1000, where MW=299.22 g/mol).* The measured values (nM) were multiplied by a factor of 7.67 to correct for dilution of the dialysate (1 part dialysate to 6.67 parts carrier plus quench).
2. Time relative to RE-024 dose: Time is the end time of the 30-min dialysate collection period.
3. BQL values were omitted from calculation of the mean.

BQL=2.56 nM

**Concentrations of PA in striatal dialysate after a single oral dose of fosmetpantotenate at 700 mg/kg in non-fasted male C57/Bl6 mice**

PA Concentration (nM) in Mouse Striatal Dialysate (Corrected for Dilution) ^(a)^

| Collection Time  (hr) ^(b)^ | MD02-M14-0405 | MD02-M14-0406 | MD02-M14-0407 | MD02-M14-0408 | MD02-M14-0409 | MD02-M14-0410 | MD02-M14-0411 | MD02-M14-0412 | Mean ^(c)^ | SD |
| --- | --- | --- | --- | --- | --- | --- | --- | --- | --- | --- |
| 0 | BQL | 52.8 | 62.6 | 44.8 | 41.3 | BQL | 51.8 | BQL | 50.7 | 8.2 |
| 0.5 | BQL | 65.1 | 65.8 | 49.7 | 41.6 | BQL | 66.5 | BQL | 57.7 | 11.4 |
| 1 | BQL | 77.7 | 78.4 | 61.6 | 60.2 | BQL | 100.1 | BQL | 75.6 | 16.2 |
| 1.5 | BQL | 46.2 | 85 | 67.5 | 52.5 | 18.9 | 99 | BQL | 61.5 | 28.7 |
| 2 | BQL | 83.3 | 154.3 | 54.6 | 73.1 | 23.2 | 95.5 | BQL | 80.7 | 44 |
| 2.5 | BQL | 86.4 | 130.1 | 55.3 | 103.9 | 19.5 | 130.1 | BQL | 87.6 | 43.8 |
| 3 | BQL | 70.3 | 209.6 | 58.4 | 84.7 | 22.4 | 126.6 | BQL | 95.3 | 65.5 |
| 3.5 | BQL | 93.1 | 227.1 | 61.6 | 97.3 | 19.9 | 190 | BQL | 114.8 | 78.6 |
| 4 | BQL | 90.3 | 239 | 81.2 | 95.5 | 23.9 | 240.4 | BQL | 128.4 | 89.9 |

1. Striatal dialysate concentrations were determined by converting measured ng/mL dialysate concentration to nM *(nM=[(ng/mL)/MW] * 1000, where MW=219.23 g/mol).* The measured values (nM) were multiplied by a factor of 7.67 to correct for dilution of the dialysate (1 part dialysate to 6.67 parts carrier plus quench).
2. Time relative to RE-024 dose: Time is the end time of the 30-min dialysate collection period.
3. BQL values were omitted from calculation of the mean.

BQL=17.49 nM

**Concentrations of fosmetpantotenate total in striatal dialysate after a single oral dose of fosmetpantotenate at 125 µg per animal in non-fasted male C57/Bl6 mice**

Fosmetpantotenate Total Concentration (nM) in Mouse Striatal Dialysate (Corrected for Dilution) ^(a)^

| Collection Time  (hr) ^(b)^ | MD02-M14-0413 | MD02-M14-0414 | MD02-M14-0415 | MD02-M14-0416 | MD02-M14-0418 | MD02-M14-0419 | MD02-M14-0420 | Mean ^(c)^ | SD |
| --- | --- | --- | --- | --- | --- | --- | --- | --- | --- |
| 0 | 130.6 | 109 | 83.3 | 310.7 | 357.9 | 216.8 | 462.4 | 238.7 | 142.9 |
| 0.5 | 27596.1 | 228593.3 | 5852246.9 | 2389398 | 1463061.7 | 6013911.1 | 2121035.3 | 2585120.3 | 2450767.6 |
| 1 | 3744.1 | 35921.8 | 1147816.4 | 370211.2 | 269332.7 | 936036.2 | 475293 | 462622.2 | 434418.3 |
| 1.5 | 245.7 | 3863.8 | 77113.9 | 16344.3 | 13224.1 | 54674.9 | 52702.6 | 31167 | 29918.7 |
| 2 | 214.2 | 1391.9 | 11639.8 | 2830.7 | 6983.9 | 18381.2 | 13579.8 | 7860.2 | 6880.6 |
| 2.5 | 209.4 | 462.4 | 4041.6 | 953.8 | 6806.1 | 14469 | 7889.2 | 4975.9 | 5197.7 |
| 3 | 166.4 | 283.9 | 1986.9 | 1092.9 | 4140.2 | 11268 | 3616.4 | 3222.1 | 3867.9 |
| 3.5 | 130.5 | 233.1 | 1705.6 | 669.3 | 3257.5 | 11914.7 | 1917.3 | 2832.6 | 4153.5 |
| 4 | 138.7 | 217.1 | 1412.9 | 549.7 | 2264.9 | 9893.9 | 1509.9 | 2283.9 | 3443.6 |

1. Striatal dialysate concentrations were determined by converting measured ng/mL dialysate concentration to nM *(nM=[(ng/mL)/MW] * 1000, where MW=474.44 g/mol).* The measured values (nM) were multiplied by a factor of 7.67 to correct for dilution of the dialysate (1 part dialysate to 6.67 parts carrier plus quench)
2. Time relative to fosmetpantotenate dose: Time is the end time of the 30-min dialysate collection period.

Concentrations were BQL in all samples for both fosmetpantotenate D1 (BQL=4.72 nM) and fosmetpantotenate D2 (BQL=3.36 nM), resulting in a BQL of
8.08 nM for fosmetpantotenate

**Concentrations of PPA total in striatal dialysate after a single oral dose of fosmetpantotenate at 125 µg per animal in
non-fasted male C57/Bl6 mice**

|  | PPA Total Concentration (nM) in Mouse Striatal Dialysate  (Corrected for Dilution) ^(a)^ | | | | | | |  |  |
| --- | --- | --- | --- | --- | --- | --- | --- | --- | --- |
| Collection Time  (hr) ^(b)^ | MD02-M14-0413 | MD02-M14-0414 | MD02-M14-0415 | MD02-M14-0416 | MD02-M14-0418 | MD02-M14-0419 | MD02-M14-0420 | Mean ^(c)^ | SD |
| 0 | BQL | BQL | BQL | 19 | 7.9 | BQL | 13.9 | 13.6 | 5.6 |
| 0.5 | 78.2 | BQL | 27.4 | 9.5 | BQL | 14.2 | BQL | 32.3 | 31.5 |
| 1 | 221.7 | 64.9 | 671.6 | 469.1 | 84.3 | 466.5 | 139.7 | 302.5 | 233.8 |
| 1.5 | 234.3 | 266.6 | 3255.4 | 1927.6 | 356.3 | 2768.4 | 1004.8 | 1401.9 | 1257.1 |
| 2 | 94.1 | 320.4 | 3434.9 | 1845.6 | 381.9 | 4127 | 1435.5 | 1662.8 | 1591.7 |
| 2.5 | 93.8 | 234.8 | 5459.9 | 1779 | 402.4 | 3563 | 2137.8 | 1953 | 1990.3 |
| 3 | 64.9 | 266.6 | 3768.1 | 2640.2 | 464 | 4178.2 | 1679 | 1865.8 | 1701.3 |
| 3.5 | 58.4 | 302.5 | 3819.4 | 1802 | 451.1 | 3563 | 1466.2 | 1637.5 | 1538.8 |
| 4 | 51.8 | 338.4 | 4152.6 | 2414.7 | 333.2 | 2496.7 | 1171.4 | 1565.5 | 1513.9 |

1. Striatal dialysate concentrations were determined by converting measured ng/mL dialysate concentration to nM *(nM=[(ng/mL)/MW] * 1000, where MW=299.22 g/mol).* The measured values (nM) were multiplied by a factor of 7.67 to correct for dilution of the dialysate (1 part dialysate to 6.67 parts carrier plus quench)
2. Time relative to RE-024 dose: Time is the end time of the 30-min dialysate collection period.
3. BQL values were omitted from calculation of the mean.

BQL=2.56 nM

**Concentrations of PA total in striatal dialysate after a single oral dose of fosmetpantotenate at 125 µg per animal in non-fasted male C57/Bl6 mice**

|  | PA Total Concentration (nM) in Mouse Striatal Dialysate  (Corrected for Dilution) ^(a)^ | | | | | | |  |  |
| --- | --- | --- | --- | --- | --- | --- | --- | --- | --- |
| Collection Time  (hr) ^(b)^ | MD02-M14-0413 | MD02-M14-0414 | MD02-M14-0415 | MD02-M14-0416 | MD02-M14-0418 | MD02-M14-0419 | MD02-M14-0420 | Mean ^(c)^ | SD |
| 0 | BQL | BQL | 25.6 | BQL | BQL | 20.9 | BQL | 23.3 | ND |
| 0.5 | BQL | BQL | BQL | BQL | BQL | BQL | BQL | BQL | ND |
| 1 | 18.2 | BQL | 58.8 | 53.5 | BQL | 34.8 | BQL | 41.3 | 18.6 |
| 1.5 | BQL | 28 | 171.8 | 126.3 | 18.4 | 185.4 | 71.7 | 100.3 | 71.8 |
| 2 | BQL | 29.6 | 374.4 | 184 | 24 | 269.4 | 122.5 | 167.3 | 137.9 |
| 2.5 | BQL | BQL | 549.3 | 167.2 | 29.5 | 348.8 | 156.7 | 250.3 | 202.2 |
| 3 | BQL | 22.9 | 437.3 | 282.7 | 39.2 | 313.5 | 153.2 | 208.1 | 164.3 |
| 3.5 | BQL | 37.8 | 465.3 | 234.1 | 48.3 | 374.4 | 181.2 | 223.5 | 172.2 |
| 4 | BQL | 45.8 | 871.2 | 465.3 | 38.8 | 398.8 | 241.8 | 343.6 | 312.4 |

1. Striatal dialysate concentrations were determined by converting measured ng/mL dialysate concentration to nM *(nM=[(ng/mL)/MW] * 1000, where MW=219.23 g/mol).* The measured values (nM) were multiplied by a factor of 7.67 to correct for dilution of the dialysate (1 part dialysate to 6.67 parts carrier plus quench).
2. Time relative to RE-024 dose: Time is the end time of the 30-min dialysate collection period.
3. BQL values were omitted from calculation of the mean.

BQL=17.49 nM

**Concentrations of fosmetpantotenate in blood after a single oral dose of fosmetpantotenate at 100 mg/kg in cynomolgus monkeys**

1. Collection Time: relative to fosmetpantotenate dose.

LLOQ blood: D1=0.86 nM, D2=0.65 nM

**Concentrations of PPA in blood after a single oral dose of fosmetpantotenate at 100 mg/kg in cynomolgus monkeys**

| Metabolite Monitored | **PPA** | | | |
| --- | --- | --- | --- | --- |
| Animal # | Cyno #1 (BB169H) | Cyno #2 (BB69J) | Cyno #3 (BA955F) | Cyno #4 (BB276H) |
| Collection Time ^(a)^ | **Blood concentration (nM)** | | | |
| -1.0 | <LLOQ | <LLOQ | <LLOQ | <LLOQ |
| 0.0 | <LLOQ | <LLOQ | <LLOQ | <LLOQ |
| 1.0 | 65.7 | 103.0 | 27.4 | 49.7 |
| 2.0 | 85.0 | 141.0 | 81.4 | 107.0 |
| 3.0 | 180.0 | 157.0 | 84.8 | 93.8 |

1. Collection Time: relative to RE-024 dose.

LLOQ: PPA=15 nM

**Concentrations of PA in blood after a single oral dose of fosmetpantotenate at 100 mg/kg in cynomolgus monkeys**

1. Collection Time: relative to fosmetpantotenate dose.

LLOQ: PA=30 nM

**Concentrations of fosmetpantotenate in intrastriatal dialysate after a single oral dose of fosmetpantotenate at 100 mg/kg in cynomolgus monkeys**

1. The reported concentrations (nM) were multiplied by a factor of 7.67 to correct for dilution of the dialysate.
2. Collection Time: relative to RE-024 dose: Time is the end time of the 30 min dialysate collection period.

LLOQ dialysate (DF adjusted): fosmetpantotenate D1=0.109 nM, fosmetpantotenate D2=0.082 nM

**Concentrations of PPA in intrastriatal dialysate after a single oral dose of fosmetpantotenate at 100 mg/kg in cynomolgus monkeys**

| Metabolite Monitored | **PPA** | | | |
| --- | --- | --- | --- | --- |
| Animal # | Cyno #1 (BB169H) | Cyno #2 (BB69J) | Cyno #3 (BA955F) | Cyno #4 (BB276H) |
| Collection Time ^(a)^ | **Dialysate concentration (nM) adjusted for dilution factor ^(b)^** | | | |
| -1.0 | 88.0 | 123.0 | 20.1 | 48.3 |
| -0.5 | 34.9 | 98.0 | 17.1 | 28.3 |
| 0.0 | 29.4 | 73.0 | 13.5 | 21.6 |
| 0.5 | 89.4 | 65.9 | 177.0 | 92.2 |
| 1.0 | 231.0 | 78.9 | 441.0 | 515.0 |
| 1.5 | 253.0 | 108.0 | 427.0 | 754.0 |
| 2.0 | 204.0 | 115.0 | 366.0 | 791.0 |
| 2.5 | 174.0 | 119.0 | 410.0 | 817.0 |
| 3.0 | 166.0 | 117.0 | 414.0 | 693.0 |

1. Collection Time: relative to fosmetpantotenate dose: Time is the end time of the 30-min dialysate collection period.
2. The reported concentrations (nM) were multiplied by a factor of 7.67 to correct for dilution of the dialysate during collection.

LLOQ dialysate (adjusted for DF): PPA=3 nM

**Concentrations of PPA in intrastriatal dialysate after a single oral dose of fosmetpantotenate at 100 mg/kg in cynomolgus monkeys**

| Metabolite Monitored | **PA** | | | |
| --- | --- | --- | --- | --- |
| Animal # | Cyno #1 (BB169H) | Cyno #2 (BB69J) | Cyno #3 (BA955F) | Cyno #4 (BB276H) |
| Collection Time ^(a)^ | **Dialysate concentration (nM) adjusted for dilution factor ^(b)^** | | | |
| -1.0 | 649 | 761 | 62 | 289 |
| -0.5 | 667 | 729 | 46 | 256 |
| 0.0 | 691 | 675 | 43 | 240 |
| 0.5 | 747 | 690 | 116 | 285 |
| 1.0 | 837 | 726 | 212 | 648 |
| 1.5 | 862 | 811 | 230 | 937 |
| 2.0 | 951 | 779 | 281 | 895 |
| 2.5 | 940 | 850 | 234 | 739 |
| 3.0 | 861 | 863 | 216 | 683 |

1. Collection Time: relative to fosmetpantotenate dose: Time is the end time of the 30 min dialysate collection period.
2. The reported concentrations (nM) were multiplied by a factor of 7.67 to correct for dilution of the dialysate during collection.

LLOQ dialysate (adjusted for DF): PA=3 nM

**Concentrations of fosmetpantotenate, PPA and PA in blood after a single oral dose of fosmetpantotenate at 300 mg/kg in cynomolgus monkeys**

| Species |  | Cyno | | | | | | |
| --- | --- | --- | --- | --- | --- | --- | --- | --- |
| Dose |  | Fosmetpantotentate 300 mg/kg PO | | | | | | |
| Analyte |  | Fosmetpantotenate | | PPA | | PA | | |
|  |  | *Cyno #1 (BB169H)* | *Cyno #2 ( BB69J)* | *Cyno #1 (BB169H)* | *Cyno #2 ( BB69J)* | *Cyno #1 (BB169H)* | | *Cyno #2 ( BB69J)* |
| Time (hr) relative to dose ^(a)^ | | Blood Concentration (nM) | | | | | | |
| Pre-dose | -1 | <LLOQ | <LLOQ | <LLOQ | <LLOQ | 139 | 251 | |
|  | 0 | <LLOQ | <LLOQ | <LLOQ | <LLOQ | 106 | 238 | |
| Post-dose | 1 | 7392.00 | 4687.62 | 876.0 | 1980.00 | 4332 | 4584 | |
|  | 2 | 1026.66 | 312.23 | 1158.0 | 2526.00 | 4176 | 3348 | |
|  | 3 | 227.06 | 183.68 | 2928.0 | 2880.00 | 3432 | 2886 | |

1. Collection time: relative to fosmetpantotenate dose.

**Concentrations of fosmetpantotenate, and PA in dialysate after a single oral dose of fosmetpantotenate at 300 mg/kg in cynomolgus monkeys**

| Species |  | Cyno | | | |
| --- | --- | --- | --- | --- | --- |
| Dose |  | Fosmetpantotentate 300 mg/kg PO | | | |
| Analyte |  | Fosmetpantotenate | | Total PA ^(a)^ | |
|  |  | *Cyno #1 (BB169H)* | *Cyno #2 ( BB69J)* | *Cyno #1 (BB169H)* | *Cyno #2 ( BB69J)* |
| Time (hr) relative to dose ^(b)^ | | *Dialysate Concentration ^(c)^* | | | |
| Dialysate pre-dose | -1 | <LLOQ | <LLOQ | 708.34 | 1218.89 |
|  | -0.5 | <LLOQ | <LLOQ | 739.00 | 1103.90 |
|  | 0 | <LLOQ | <LLOQ | 850.93 | 1042.58 |
| Dialysate post-dose | 0.5 | 2055.87 | 1059.88 | 705.27 | 1303.22 |
|  | 1 | 393.02 | 429.27 | 1103.90 | 2039.16 |
|  | 1.5 | 76.05 | 72.40 | 1349.22 | 1962.50 |
|  | 2 | 20.01 | 19.06 | 1786.18 | 2284.47 |
|  | 2.5 | 7.12 | 4.54 | 1870.50 | 2269.14 |
|  | 3 | BLQ | 23.70 | 2207.81 | 2138.81 |

1. The reported concentration of PA has not been adjusted for baseline levels.
2. Collection Time: relative to fosmetpantotenate dose: Time is the end time of the 30 min dialysate collection period.
3. The reported concentrations (nM) were multiplied by a factor of 7.67 to correct for dilution of the dialysate during collection.

LLOQ dialysate (corrected for DF): fosmetpantotenate D1=0.2 nM, fosmetpantotenate D2=0.3 nM, PA=6 nM

BQL: below quantitation limit; DF: dilution factor; LLOQ: lower limit of quantitation; ND: not determined; PA: pantothenate; PPA: phosphopantothenate;
RE-024: fosmetpantotenate; SD: standard deviation
