# Supplementary material for: Fosmetpantotenate (RE-024), a phosphopantothenate replacement therapy for pantothenate kinase-associated neurodegeneration: Mechanism of action and efficacy in nonclinical models
Source: PLoS One. 2018 Mar 9;13(3):e0192028. doi: 10.1371/journal.pone.0192028 (PMC5844530; doi:10.1371/journal.pone.0192028)
Supplement: S7 Table — (DOCX) [file pone.0192028.s009.docx]

**S7 Table.** **Figure 3 (Main Manuscript) Data.**

|  | Area Ratio | | CoASH Fold Increase | | ANOVA (over vehicle) |
| --- | --- | --- | --- | --- | --- |
| Concentration (µM) | Mean | SD | Mean | SD |  |
| vehicle | 0.055 | 0.001 | 1.00 | 0.051 | - |
| 12.5 | 0.081 | 0.003 | 1.06 | 0.075 | n.s. |
| 25 | 0.111 | 0.003 | 1.25 | 0.057 | n.s. |
| 50 | 0.141 | 0.009 | 2.00 | 0.183 | p<0.001 |
| 100 | 0.127 | 0.007 | 2.27 | 0.339 | p<0.0001 |
| 200 | 0.216 | 0.013 | 2.41 | 0.282 | p<0.0001 |

ANOVA: analysis of variance; CoASH: reduced coenzyme A; SD: standard deviation

|  |  | Area Ratio | | |
| --- | --- | --- | --- | --- |
|  |  | Biological replicate 1 | Biological replicate 2 | Biological replicate 3 |
| Compound conc (µM) | PANK2-/- vehicle | 0.0691 | 0.0635 | 0.0635 |
| 200 | Fosmetpantotenate | 0.243 | 0.294 | 0.241 |
| 100 | Fosmetpantotenate | 0.157 | 0.186 | 0.139 |
| 50 | Fosmetpantotenate | 0.159 | 0.189 | 0.167 |
| 25 | Fosmetpantotenate | 0.141 | 0.129 | 0.135 |
| 12.5 | Fosmetpantotenate | 0.109 | 0.098 | 0.112 |
