# Supplementary material for: Fosmetpantotenate (RE-024), a phosphopantothenate replacement therapy for pantothenate kinase-associated neurodegeneration: Mechanism of action and efficacy in nonclinical models
Source: PLoS One. 2018 Mar 9;13(3):e0192028. doi: 10.1371/journal.pone.0192028 (PMC5844530; doi:10.1371/journal.pone.0192028)
Supplement: S8 Table — (DOCX) [file pone.0192028.s010.docx]

**S8 Table. Figure 4 (Main Manuscript) Data.**

CoASH and Total CoA

|  | **CoASH** | | |  |  | **Total CoA** | | |
| --- | --- | --- | --- | --- | --- | --- | --- | --- |
|  | ***Biological replicates**** | | |  |  | ***Biological replicates**** | | |
|  | Area ratio | | |  |  | Area ratio | | |
| Day 1 – 1 µM fosmetpantotenate | 0.0013 | 0.0013 | 0.0014 |  | Day 1 – 1 µM fosmetpantotenate | 0.0238 | 0.0248 | 0.0256 |
|  | 0.0011 | 0.0012 | 0.0012 |  |  | 0.0250 | 0.0256 | 0.0258 |
|  | 0.0018 | 0.0019 | 0.0019 |  |  | 0.0229 | 0.0233 | 0.0235 |
| Day 1 – vehicle | 0.0013 | 0.0013 | 0.0013 |  | Day 1 – vehicle | 0.0139 | 0.0145 | 0.0143 |
|  | 0.0018 | 0.0018 | 0.0019 |  |  | 0.0145 | 0.0147 | 0.0147 |
|  | 0.0014 | 0.0013 | 0.0013 |  |  | 0.0136 | 0.0139 | 0.0140 |
| Day 3 – 1 µM fosmetpantotenate | 0.0035 | 0.0037 | 0.0037 |  | Day 3 – 1 µM fosmetpantotenate | 0.0360 | 0.0361 | 0.0365 |
|  | 0.0024 | 0.0025 | 0.0026 |  |  | 0.0359 | 0.0365 | 0.0368 |
|  | 0.0022 | 0.0023 | 0.0023 |  |  | 0.0185 | 0.0353 | 0.0367 |
| Day 3 – vehicle | 0.0022 | 0.0023 | 0.0022 |  | Day 3 – vehicle | 0.0132 | 0.0130 | 0.0129 |
|  | 0.0021 | 0.0022 | 0.0020 |  |  | 0.0131 | 0.0132 | 0.0133 |
|  | 0.0018 | 0.0017 | 0.0018 |  |  | 0.0125 | 0.0128 | 0.0129 |
| Day 5 – 1 µM fosmetpantotenate | 0.0029 | 0.0031 | 0.0031 |  | Day 5 – 1 µM fosmetpantotenate | 0.0314 | 0.0330 | 0.0330 |
|  | 0.0024 | 0.0024 | 0.0025 |  |  | 0.0343 | 0.0336 | 0.0342 |
|  | 0.0019 | 0.0020 | 0.0020 |  |  |  |  |  |
| Day 5 – vehicle | 0.0020 | 0.0019 | 0.0020 |  | Day 5 – vehicle | 0.0140 | 0.0132 | 0.0136 |
|  | 0.0024 | 0.0025 | 0.0025 |  |  | 0.0136 | 0.0141 | 0.0138 |
|  | 0.0014 | 0.0015 | 0.0014 |  |  | 0.0141 | 0.0140 | 0.0138 |
|  |  |  |  |  |  |  |  |  |
| Pre-dose | 0.0010 | 0.0011 | 0.0012 |  | Pre-dose | 0.0238 | 0.0192 | 0.0190 |
|  | 0.0009 | 0.0009 | 0.0007 |  |  | 0.0169 | 0.0169 | 0.0168 |
|  | 0.0018 | 0.0018 | 0.0019 |  |  | 0.0177 | 0.0179 | 0.0188 |

*Each biological replicate (well) was analyzed 3 times (analytical replicates)

CoA: coenzyme A; CoASH: reduced coenzyme A

Acetyl Tubulin Quantification

Experiment 1

|  |  |  | **Acetyl tubulin** | **Actin** | **Normalized** |
| --- | --- | --- | --- | --- | --- |
|  | T0 | Replicate 1 | 8020.347 | 4956.962 | 1.6 |
|  |  | Replicate 2 | 6970.225 | 5282.134 | 1.3 |
|  |  | Replicate 1 | 6359.811 | 7254.861 | 0.9 |
|  |  | Replicate 2 | 6140.225 | 7376.154 | 0.8 |
| ***Vehicle (0.1% DMSO)*** | DAY 1 | Replicate 1 | 7259.761 | 5669.841 | 1.3 |
|  |  | Replicate 2 | 7928.882 | 5727.376 | 1.4 |
|  | DAY 3 | Replicate 1 | 8650.64 | 6696.134 | 1.3 |
|  |  | Replicate 2 | 7349.054 | 5860.134 | 1.3 |
|  | DAY 5 | Replicate 1 | 8625.589 | 6647.134 | 1.3 |
|  |  | Replicate 2 | 9358.468 | 6829.669 | 1.4 |
| ***1 µM fosmetpantotenate*** | DAY 1 | Replicate 1 | 5352.933 | 5408.033 | 1.0 |
|  |  | Replicate 2 | 4967.69 | 5159.033 | 1.0 |
|  | DAY 3 | Replicate 1 | 5869.761 | 4810.74 | 1.2 |
|  |  | Replicate 2 | 6720.347 | 4592.74 | 1.5 |
|  | DAY 5 | Replicate 1 | 9323.933 | 4608.619 | 2.0 |
|  |  | Replicate 2 | 10851.882 | 5049.962 | 2.1 |

Experiment 2

|  |  |  | **Acetyl tubulin** | **Actin** | **Normalized** |
| --- | --- | --- | --- | --- | --- |
|  | T0 | Replicate 1 | 4444.912 | 3471.489 | 1.3 |
|  |  | Replicate 2 | 4808.782 | 2769.426 | 1.7 |
|  |  | Replicate 1 | 4199.033 | 3207.083 | 1.3 |
|  |  | Replicate 2 | 3571.79 | 3106.569 | 1.1 |
| ***Vehicle (0.1% DMSO)*** | DAY 1 | Replicate 1 | 3834.64 | 2995.326 | 1.3 |
|  |  | Replicate 2 | 3525.539 | 2183.87 | 1.6 |
|  | DAY 3 | Replicate 1 | 3598.175 | 2074.355 | 1.7 |
|  |  | Replicate 2 | 4067.326 | 3336.083 | 1.2 |
|  | DAY 5 | Replicate 1 | 3411.719 | 2769.861 | 1.2 |
|  |  | Replicate 2 | 3892.64 | 3012.255 | 1.3 |
| ***1 µM fosmetpantotenate*** | DAY 1 | Replicate 1 | 2820.548 | 2488.811 | 1.1 |
|  |  | Replicate 2 | 2422.355 | 2436.861 | 1.0 |
|  | DAY 3 | Replicate 1 | 5646.447 | 2169.355 | 2.6 |
|  |  | Replicate 2 | 4155.426 | 1618.134 | 2.6 |
|  | DAY 5 | Replicate 1 | 4976.426 | 2220.104 | 2.2 |
|  |  | Replicate 2 | 5163.962 | 2776.861 | 1.9 |
